# Comparative efficacy, safety and cost of oral Chinese patent medicines for rheumatoid arthritis: a Bayesian network meta-analysis

**DOI:** 10.1186/s12906-020-03004-4

**Published:** 2020-07-06

**Authors:** Dan Zhang, Jin-tao Lyu, Bing Zhang, Xiao-meng Zhang, Hao Jiang, Zhi-jian Lin

**Affiliations:** Department of Clinical Chinese Pharmacy, School of Chinese Materia Medica, Beijing University of Chinese Medicine, Beijing, 102488 China

**Keywords:** Chinese patent medicines, Rheumatoid arthritis, Safety, Efficacy, Cost, Network meta-analysis

## Abstract

**Background:**

Rheumatoid arthritis (RA) is a common inflammatory disease with a substantial burden for society and economic worldwide. Chinese patent medicines (CPMs) have gained attention as alternative remedies due to they can exert the satisfactory therapeutic effects via holistic regulation. Currently, several oral Chinese patent medicines are routinely recommended for managing and treating RA. Therefore, a network meta-analysis (NMA), which tries to synthesize evidences for a decision making by evaluating the comparative effectiveness of multiple interventions against the same disease, was undertaken to identify the optimal intervention according to their efficacy in clinical treatment and symptom remission, safety profile and daily cost.

**Methods:**

Randomized controlled trials (RCTs) regarding CPMs to treat RA were comprehensive retrieved from 3 foreign databases and 4 Chinese databases, and the retrieved results were last updated on January 10, 2019. The bias of the selected trials was assessed by two individuals independently through RoB2. A random-effects model was adopted during the meta-analytic procedures, and outcomes concerning efficacy and safety were evaluated as odds ratios (OR), mean differences (MD) and 95% credible intervals (CI) utilizing Stata 14.1 and WinBUGS 1.4.3 software. Furthermore, the cluster analysis and comprehensive investigation were preformed concerning the comparative efficacy, safety and cost of oral CPMs.

**Results:**

One hundred sixteen RCTs involving 10,213 individuals met the inclusion criteria and were enrolled into current NMA. The results from existing evidence indicated that Biqi capsule and Yuxuebi capsule probably had a favorable balance in consideration of benefits, tolerability and daily cost. Furthermore, as the least expensive choice, glucosides of Tripterygium Wilfordii tablet was associated with displaying a trend of relieving joint tenderness, joint swelling, and morning stiffness for patients with RA.

**Conclusion:**

Biqi capsule, Yuxuebi capsule and glucosides of Tripterygium Wilfordii tablet were recommended for treating RA based on the favorable benefits in both clinical efficacy and symptoms, and they, meanwhile, might be associated with the more tolerable and acceptable therapeutic alternative in terms of safety profile and daily cost. Nevertheless, the additional results from high-quality, multi-center and head-to-head trials would be pivotal for supporting our findings.

## Background

Rheumatoid arthritis (RA) is a chronic inflammatory joint disease with a global prevalence of an estimated 0.2–1.0%, which can cause cartilage and bone damage as well as disability, and even will affect working efficiency, performance status, life expectancy, and produce the negative thoughts for patients [1–4]. RA generates a heavy socioeconomic burden, and overall well-being such as emotional and physical components may vary in individuals [5, 6]. Substantial epidemiological evidence indicates that persistent systemic inflammation and immune dysfunction may be viewed as clinically important risk factors for various diseases or co-morbidities, especially cardiovascular diseases, osteoporosis, interstitial lung disease and malignancies [7–9]. The past decades have brought important advances in the understanding of rheumatoid arthritis and its management. Biologic disease-modifying anti-rheumatic drugs (DMARDs) and nonsteroidal anti-inflammatory drugs (NSAIDs) have been introduced that can ameliorate the signs and symptoms, and modify the irregular immune response [10–12]. Nevertheless, many patients still have an unpleasant experience due to unwanted adverse drug reactions (ADRs). Accordingly, the novel efficacious agents and pharmacological strategies for RA treatment are urgently required [13–15].

To this relevant issue, traditional Chinese medicine (TCM) has such unique advantages as multiple pathways, multi-targets to holistic treatment against RA, and an essential component of the current medical system, TCM has been extensively used in clinical practice for thousands of years in Asian countries [16, 17]. RA falls into the category of *Bi* symptoms with complex impedimental conditions and deficiency patterns, which is caused by the invasion of wind, dampness or heat pathogens on the meridian channels into the human body [18, 19]. In the clinical practice of TCM, oral Chinese patent medicines (CPMs) from well-known and effective Chinese medicine formula after being approved by Chinese authorities for treatment of RA are easy to take which is adapted to to modern life with the recent development of pharmaceutics [20]. It has been estimated that there are dozens of CPMs on the market for the management of RA, and some of these have been tested in clinical studies and may be worthy of consideration for RA treatment [21, 22].

While the optimal choice of CPMs against RA in clinical practice is unclear, their comparative efficacy, safety and cost also remains inconclusive. To overcome the restrictions of limited available comparisons, this research employed a Bayesian network meta-analysis (NMA) and hierarchical cluster analysis to comprehensively compare and rank the efficacy, safety and cost of these available oral CPMs for strengthen inferences to guide and support decision making in the field of TCM in rheumatology.

## Methods

The procedure of current NMA was conducted in accordance with the Preferred Reporting Items for Systematic reviews and Meta-Analyses (PRISMA) guidelines “NMA extended version” [23]. The completed PRISMA check list was presented as **Supplementary file** **1**. The present NMA did not require ethical approval because it gathered data from trials that retrieved from public databases.

### Literature search strategy

In this network meta-analysis, the potentially eligible RCTs regarding CPMs to treat RA were recruited through the electronic databases of Embase, PubMed, Cochrane Library, the China National Knowledge Infrastructure Database (CNKI), the Wan-fang Database, the Cqvip Database (VIP), and the China Biology Medicine disc (CBMdisc) from inception of database construction’ s date to October 3, 2018. The retrieved results were last updated on January 10, 2019. To identify relevant publications, searching terms were constructed following the Cochrane systematic review methodology [24]. The following searching terms of RA were adopted: “Arthritis, Rheumatoid [MeSH Terms]”, “Rheumatoid Arthritis”, “Rheumatoid”, “Arthritis”, and “Rheumatoid Nodule”. Besides, the searched CPMs were all listed in *the Catalogue of Drugs for Basic National Medical Insurance* (The 2017 Edition) [25]. The retrieval strategies were translated into syntax appropriate for different electronic databases. Further eligible studies were sought by manually searching from the reference lists of relevant meta-analyses and the retrieved review articles. Additionally, the complete search strategy was provided in the **Supplementary file** **2**. In addition, there was no limitation on publication year, language, and blinding methods. More details about the product information (raw materials, labeled efficacy, indications) of CPMs were presented in **Supplementary Table** **1** to facilitate rheumatologists and pharmacologists to acquire the comprehensive information of the included CPMs.

### Selection criteria

Inclusion and exclusion criteria were discussed and defined a priori by consensus according to the PICOS (patients, intervention, comparison, outcome, and study design) framework. Two investigators independently perused the titles and abstracts of the identified RCTs, the trials enrolled in present NMA meet the following criteria: (1) study design should be RCT; (2) enrolled patients suffered from RA, without limitations on gender, age or nationality of participants, course and progression of disease; (3) the intervention should be oral CPMs, and the selected oral CPMs were all listed in *the Catalogue of Drugs for Basic National Medical Insurance* (The 2017 Edition) [25], those patients were allocated to oral CPMs receiving the monotherapy of CPMs or in combination with conventional medicines (CM); accordingly, a control for CM group was treated by conventional medicines alone; (4) the primary outcomes included the clinical effectiveness rate and the incidence of ADRs, the clinical effectiveness rate were defined by t*he American College of Rheumatology (ACR)* core set of disease activity measures [26], the definition of the clinical effectiveness rate was mainly consistent with ACR guidelines; and the secondary outcomes covered joint tenderness, joint swelling, morning stiffness, and erythrocyte sedimentation rate (ESR).

Trials were excluded as follows: (1) quasi-randomized trials, abstracts, reviews, case reports, comments, editorials, and pharmacological experiments, duplication, and unavailable articles, etc. (2) the subjects with serious liver and kidney dysfunction; (3) the information of medication, such as dosage, route of administration, course for interventions and comparisons was insufficient. The included interventions could not provide connections through the network meta-analysis. The treatment approaches contained surgery, injection, TCM decoction, acupuncture, massage or other local therapies. Furthermore, the intervention that could not generate the closed loop with other comparisons, or only included in one trail was excluded to avoid essentially the small size samples problem. (4) The trial did not report the efficacy or safety outcomes.

### Data extraction and quality assessment

Using a standardized data abstraction sheet in Microsoft Excel (Microsoft Corp, Redmond, WA), two investigators independently extracted the corresponding data for included trial. The following information were recorded from the included RCTs: characteristics of the enrolled patients with RA, for instance, sample size, age, gender, race, diagnostic criteria, course and severity of disease. The intervention information involved the names, dosages, duration of oral CPMs and conventional medicine.

The quality assessment of individual trial were completed by using Revised Cochrane risk-of-bias tool for randomized trials (RoB 2) according to the Cochrane risk-of-bias tool (*Cochrane Handbook for Systematic Reviews of Interventions*, version 6.0) including the following 5 domains: (1) Randomization process, (2) Deviations from intended interventions, (3) Missing outcome data, (4) Measurement of the outcome, (5) Selection of the reported result, and based on these, an overall risk-of-bias judgement was reached for a result [27]. Each of these domains was categorized as three levels as high, low or some concerns. Any disagreement was resolved through discussion of the two assessors or, where necessary, in consultation with a third investigator.

### Statistical analysis

The mean differences (MD) were chosen as the effect sizes for continuous results, while dichotomous results were calculated using pooled odds ratios (OR) with corresponding 95% confidence intervals (CI). Given the clinical and methodological heterogeneity of both methods and participants among the enrolled trials, the random-effects model was selected during statistical processes [28, 29]. First, the Bayesian NMA through the Marko chain Monte Carlo (MCMC) method was performed by utilizing WinBUGS1.4.3 software (version 1.4.3; MRC Biostatistics Unit, Cambridge, UK). The posterior samples and analysis procedure of MCMC simulation were generated as 200,000 iterations to produce the outputs, the first 10,000 burn-in iterations to allow convergence and annealing algorithm [30]. Meanwhile, the hierarchy of treatment rankings was estimated by the value of surface under the cumulative ranking curve (SUCRA), and the larger SUCRA value of comparisons was regarded as the superior efficacy or the lower the toxicity [31, 32]. In addition, Stata software (version 14.1; Stata Corp, College Station, TX) was undertaken to depict relevant diagrams of current NMA. The network graph could display the relationship among different interventions for each outcome, the node sizes indicated total sample sizes for treatments, and line thicknesses corresponded to the number for trials [33–35]. The inconsistency in each closed loop was evaluated using the loop-specific approach to evaluate the agreement between direct and indirect sources of evidence, and it was regarded as a better consistency when the lower bound of and their 95% CIs for the inconsistency factors (IF) was equal to zero [36–38]. In terms of the publication bias and small-size effects of included RCTs for the clinical effectiveness rate, it was graphically accessed via a comparison-adjusted funnel plot, and Egger’s test were applied to measure the asymmetry; the results of Egger test (*P* > .05) were defined as non-significant publication bias among included RCTs [39]. Besides, the cluster analysis was conducted for identifying the optimal treatments under two independent endpoints simultaneously [40].

Correspondingly, Origin software was employed to present the stereo histogram for comprehensive investigating the comparative efficacy, safety and cost of oral CPMs against RA. The SUCRA values about the clinical effectiveness rate and the incidence of ADRs were represented efficacy and safety, respectively. Reference prices of all available oral CPMs were obtained from jianke.com (http://www.jianke.com) that was a legitimate online pharmacy certified by the National Medical Products Administration in China, the daily drug cost were exclusive of indirect cost.

## Results

### Study selection and characteristics

**Fig. 1:**
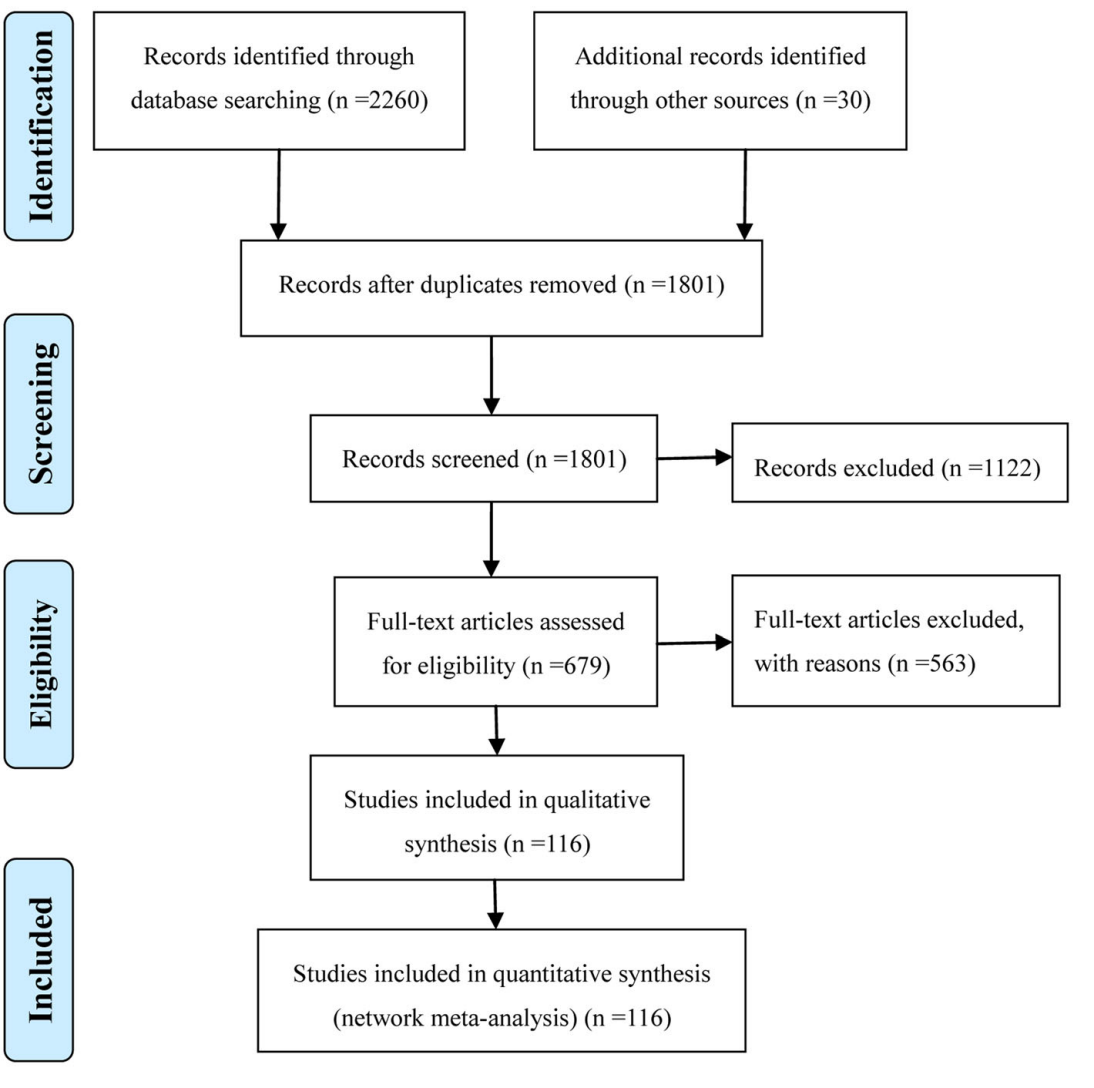
Flow diagram of the search for eligible studies

The selection process was illustrated in Fig. 1, a total of 2290 publications were identified through electronic and manual searches as mentioned. After the initial screening to exclude the irrelevant and duplicate articles, 679 potentially eligible trials were detailed assessed of the full text. Eventually, 116 RCTs involving 10,213 subjects met the inclusion criteria and were selected into present NMA from 2000 to 2018. Among them, 30 RCTs were acquiring from manual searches, and 3 RCTs were published as English-language articles (**Supplementary file** **3**). In addition, this NMA incorporated 11 CPMs, the full name, abbreviation, and trials’ number of each CPM was listed in Table 1.

**Table 1:** The full name, abbreviation, and trials’ number of included CPMs

Full name of CPMs	Abbreviation of CPMs	The trials’ number of each CPMs
Biqi capsule	BQ	11
Fufang-Fengshining capsule/ tablet	FufangFSN	5
Jingulian capsule	JGL	2
Kunxian capsule	KX	9
glucosides of Tripterygium Wilfordii tablet	GTW	46
Leigongteng tablet	LGT	4
Qiweitongbi oral liquid	QWTB	2
Wangbi tablet	WB	3
Yishenjuanbi pill	YSJB	10
Yuxuebi capsule/ tablet	YXB	2
Zhengqingfengtongning tablet/ sustained-release tablet/ capsule	ZQFTN	38

**Fig. 2:**
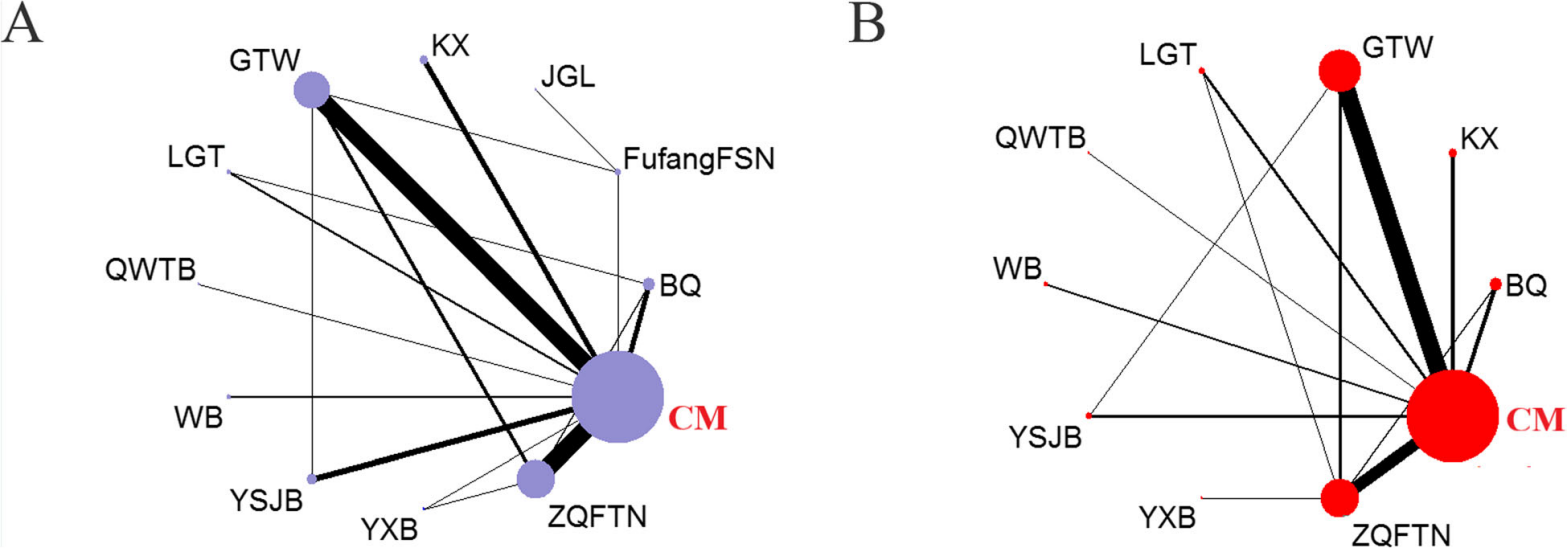
The evidence network of all enrolled RCTs about different CPMs. Note: **a**: the clinical effectiveness rate; **b**: the incidence of ADRs. Node sizes indicated total sample sizes for treatments. Line thicknesses corresponded to the number for trials

Overall, a total of 10,213 patients with RA were enrolled in these studies, and all these study objects were from Asian populations. Totally, 5941 cases were allocated to CPM group (2337 cases used CPM monotherapy, and 3604 adopted combination therapy of CPM plus CM), and on the contrary, 4272 patients received CM alone. The 111 RCTs reported the information of gender, female patients accounted for 68.14% approximately, and the majority of subjects were middle-aged and elderly. Apart from this, information on age range was available from 79 (68.10%) of 116 records with the mean or median age ranging from ranged from 11 to 81 years old, and the sample size in the trails with disparities varied from 22 to 236. The duration in most RCTs were administering the treatment for 14 days. Conventional medicine covered methotrexate, leflunomide, meloxicam, celecoxib, loxoprofen sodium, nimesulide, naproxen, indomethacin, aceclofenac acid, diclofenac sodium, oxaprozin, sulfasalazine, etc.. The baseline characteristics and reference list of the included RCTs were summarized in Supplementary **Table** **2**. The entire network plots of different comparisons for primary outcomes were demonstrated in Fig. 2.

### Assessment of methodological quality

**Fig. 3:**
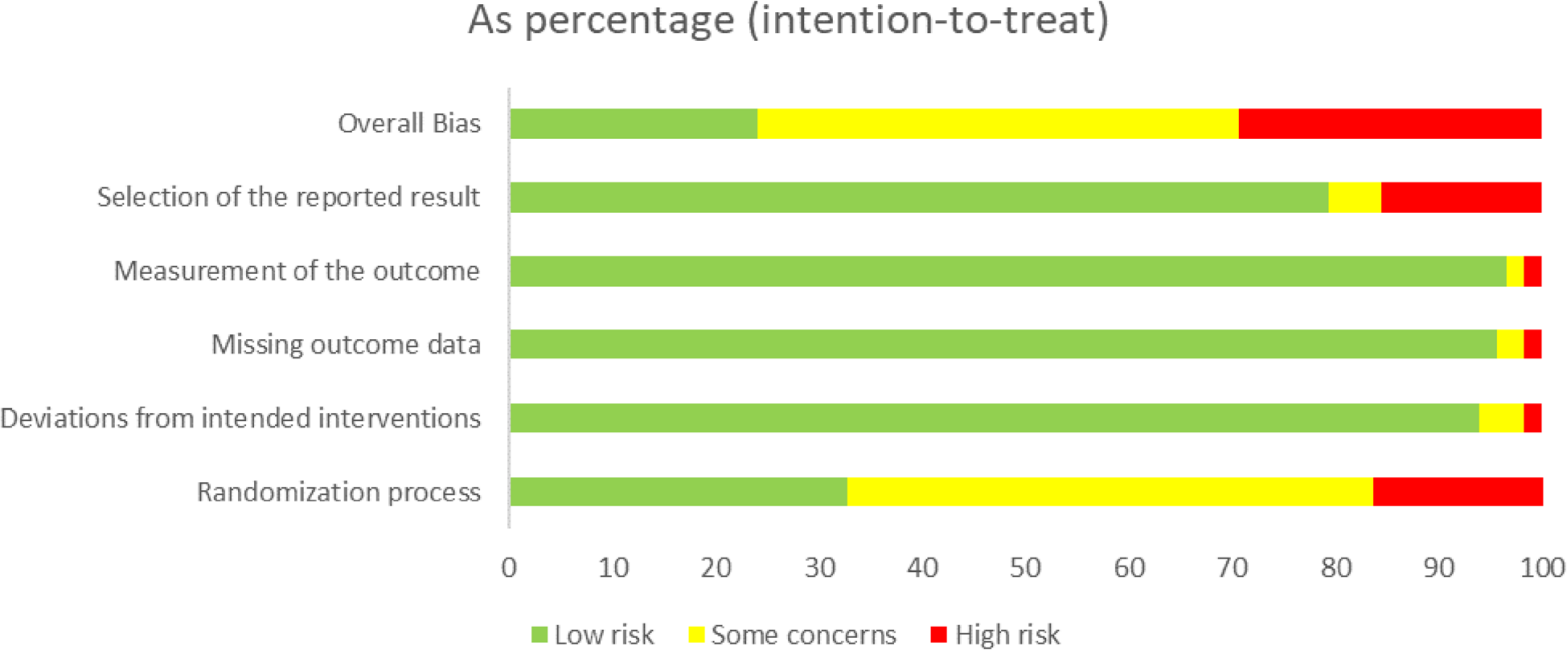
Risk-of-bias graph

The risk-of-bias graph was indicated in Fig. 3 and more details were shown in **Supplementary Table** **5****.** For randomization process domain, 38 RCTs were rated as low risk of bias, among which only 4 described adequate allocation concealment. 109 RCTs and 111 RCTs had a low risk of bias in deviations from intended interventions and missing outcome data, respectively. Moreover, of these included trials, 112 trials and 92 trials were prone to a low risk of bias regarding measurement of the outcome and selection of the reported result domain. Overall, 28 trials were deemed as low risk of bias, showing the moderate quality of selected RCTs.

### The clinical effectiveness rate

The clinical effectiveness rate was available for 11 different oral CPMs from the 97 studies. According to the results of NMA that illustrated in Table 2, some CPMs involving Biqi capsule (BQ) (OR = 0.22, 95%CI: 0.13–0.39), Fufang-Fengshining capsule (FufangFSN) (OR = 0.15, 95%CI: 0.042–0.47), Kunxian capsule (KX) (OR = 0.39, 95%CI: 0.20–0.77), glucosides of Tripterygium Wilfordii tablet (GTW) (OR = 0.64, 95%CI: 0.46–0.87), Yishenjuanbi pill (YSJB) (OR = 0.19, 95%CI: 0.098–0.36), Yuxuebi tablet (YXB) (OR = 0.27, 95%CI: 0.084–0.83), Zhengqingfengtongning tablet (ZQFTN) (OR = 0.28, 95%CI: 0.20–0.38) monotherapy or in combination with CM could appear to be a promising strategy for improving the clinical effectiveness rate in comparison to CM alone, and significant difference was found between groups.

**Table 2:** Results of the network meta-analysis for the clinical effectiveness rate (upper-right quadrant) and the incidence of ADRs (lower-left quadrant)

BQ	1.54 (0.42,6.03)	0.28 (0.033,2.31)	0.58 (0.25,1.35)	0.35 (0.19,0.66)	0.27 (0.10,0.67)	0.57 (0.10,3.13)	0.55 (0.15,2.04)	1.17 (0.50,2.79)	0.85 (0.24,2.98)	0.82 (0.45,1.48)	0.22 (0.13,0.39)
–	**FufangFSN**	**0.19 (0.032,0.92)**	0.38 (0.095,1.43)	**0.23 (0.065,0.75)**	**0.17 (0.038,0.74)**	0.37 (0.049,2.74)	0.36 (0.066,1.90)	0.76 (0.19,2.94)	0.55 (0.10,2.81)	0.53 (0.15,1.78)	**0.15 (0.042,0.47)**
–	–	**JGL**	2.05 (0.25,18.11)	1.25 (0.16,10.08)	0.95 (0.10,8.95)	2.02 (0.15,28.02)	1.94 (0.19,21.21)	4.14 (0.49,37.31)	2.99 (0.29,31.93)	2.88 (0.37,23.56)	0.79 (0.10,6.38)
**2.63 (1.10,6.31)**	–	–	**KX**	0.61 (0.30,1.20)	0.46 (0.16,1.30)	0.98 (0.17,5.47)	0.94 (0.25,3.55)	2.02 (0.82,4.93)	1.45 (0.39,5.31)	1.40 (0.69,2.80)	**0.39 (0.20,0.77)**
**3.42 (1.80,6.69)**	–	–	1.30 (0.67,2.55)	**GTW**	0.76 (0.31,1.85)	1.61 (0.32,8.34)	1.55 (0.47,5.33)	**3.32 (1.65,6.85)**	2.39 (0.74,7.78)	**2.30 (1.51,3.54)**	**0.64 (0.46,0.87)**
**2.71 (1.04,7.17)**	–	–	1.03 (0.39,2.76)	0.79 (0.36,1.74)	**LGT**	2.13 (0.35,13.24)	2.04 (0.49,8.86)	**4.39 (1.52,12.82)**	3.16 (0.77,13.02)	**3.05 (1.25,7.51)**	0.84 (0.36,1.95)
1.09 (0.15,6.81)	–	–	0.41 (0.055,2.58)	0.32 (0.046,1.80)	0.39 (0.051,2.63)	**QWTB**	0.96 (0.13,6.99)	2.06 (0.36,11.69)	1.48 (0.21,10.59)	1.43 (0.28,7.31)	0.39 (0.078,1.93)
0.35 (0.070,1.40)	–	–	**0.13 (0.027,0.53)**	**0.10 (0.023,0.36)**	**0.13 (0.024,0.55)**	0.32 (0.033,3.20)	**WB**	2.14 (0.56,8.09)	1.55 (0.30,7.76)	1.49 (0.43,4.94)	0.41 (0.12,1.29)
2.22 (0.88,5.59)	–	–	0.84 (0.33,2.12)	0.65 (0.31,1.33)	0.82 (0.30,2.26)	2.04 (0.32,15.63)	**6.38 (1.55,32.01)**	**YSJB**	0.72 (0.19,2.67)	0.70 (0.33,1.43)	**0.19 (0.098,0.36)**
0.28 (0.027,1.95)	–	–	**0.11 (0.010,0.75)**	**0.083 (0.0085,0.52)**	**0.10 (0.0097,0.76)**	0.26 (0.015,3.68)	0.82 (0.063,8.61)	**0.13 (0.012,0.91)**	**YXB**	0.96 (0.31,3.04)	**0.27 (0.084,0.83)**
**2.30 (1.21,4.55)**	–	–	0.88 (0.44,1.77)	**0.68 (0.47,0.96)**	0.85 (0.38,1.93)	2.13 (0.37,14.86)	**6.63 (1.86,30.16)**	1.04 (0.49,2.24)	**8.10 (1.35,77.49)**	**ZQFTN**	**0.28 (0.20,0.38)**
**4.13 (2.27,7.71)**	–	–	1.57 (0.85,2.94)	1.21 (0.94,1.54)	1.53 (0.72,3.24)	3.82 (0.68,26.04)	**11.85 (3.46,52.11)**	1.86 (0.94,3.72)	**14.55 (2.36,140.9)**	**1.79 (1.31,2.44)**	**CM**

Note: Results in bold reached significant difference

In addition, significant differences were detected across different CPMs, FufangFSN were superior to Jingulian capsule (JGL) in improving the clinical effectiveness rate (OR = 0.19, 95%CI: 0.032–0.92). Similarly, BQ (OR = 0.35, 95%CI: 0.19–0.66), FufangFSN (OR = 0.23, 95%CI: 0.065–0.75), YSJB (OR = 3.32, 95%CI: 1.65–6.85) and ZQFTN (OR = 2.30, 95%CI: 1.51–3.54) could both achieve the better clinical effectiveness rates than GTW. BQ (OR = 0.27, 95%CI: 0.10–0.67), FufangFSN (OR = 0.17, 95%CI: 0.038–0.74), YSJB (OR = 4.39, 95%CI: 1.52–12.82) and ZQFTN (OR = 3.05, 95%CI: 1.25–7.51) could present the higher clinical effectiveness rates relative to those with Leigongteng tablet (LGT).

**Fig. 4:**
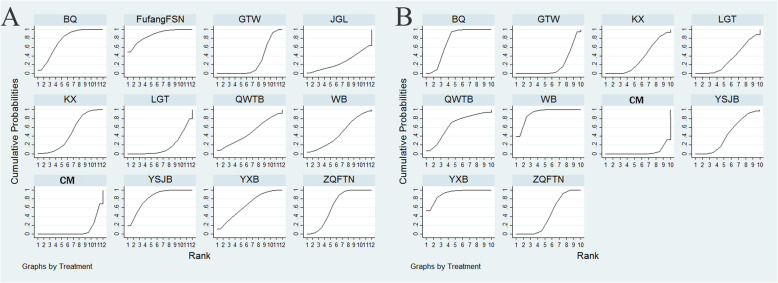
Rank of the cumulative probabilities for primary outcomes. Note: **a**: the clinical effectiveness rate; **b**: the incidence of ADRs

Corresponding, FufangFSN was associated with the highest probability of the best option for improving the clinical effectiveness rate (87.41%), followed by YSJB (82.04%) and YXB (75.57%), as shown in Fig. 4a. The SUCRA values of the other CPMs were listed in Table 3.

**Table 3:** The comparative efficacy, safety and costs of included CPMs

CPMs	The SUCRA of efficacy	The SUCRA of safety	Daily costs (RMB/day)
Yishenjuanbi pill	82.04%	44.22%	40.5
Biqi capsule	75.57%	73.58%	17.5
Yuxuebi capsule	65.68%	91.59%	22.89
Zhengqingfengtongning tablet	64.69%	44.05%	12.13
Qiweitongbi oral liquid	49.14%	65.01%	18.15
Kunxian capsule	48.49%	34.91%	63.33
Wangbi tablet	47.11%	91.05%	8.75
glucosides of Tripterygium Wilfordii tablet	27.12%	18.48%	7.56
Leigongteng tablet	17.46%	32.98%	2.09

### The incidence of ADRs

Data on the incidence of ADRs could be derived from 82 records for comparing 10 CPMs. The results in Table 2 suggested that BQ (OR = 4.13, 95%CI: 2.27–7.71), Wangbi tablet (WB) (OR = 11.85, 95%CI: 3.46–52.11), Yuexuebi capsule (YXB) (OR = 14.55, 95%CI: 2.36–140.9) and ZQFTN (OR = 1.79, 95%CI: 1.31–2.44) monotherapy or in combination with CM could both exhibit an advantage in reducing the incidence of ADRs compared with CM alone, and these between-group differences did reach statistically significance.

Concerning about the significant differences among different CPMs, WB might have the more favorable trend for relieving the incidence of ADRs than KX (OR = 0.13, 95%CI: 0.027–0.53), GTW (OR = 0.10, 95%CI: 0.023–0.36), LGT (OR = 0.13, 95%CI: 0.024–0.55), YSJB (OR = 6.38, 95%CI: 1.55–32.01) and ZQFTN (OR = 6.63, 95%CI: 1.86–30.16). Also, the superiority of YXB for reducing the incidence of ADRs over some CPMs involving KX (OR = 0.11, 95%CI: 0.010–0.75), GTW (OR = 0.083, 95%CI: 0.0085–0.52), LGT (OR = 0.10, 95%CI: 0.0097–0.76), YSJB (OR = 0.13, 95%CI: 0.012–0.91), ZQFTN (OR = 8.10, 95%CI: 1.35–77.49). In addition, BQ showed a better profile of safety compared with KX (OR = 2.63, 95%CI: 1.10–6.31), GTW (OR = 3.42, 95%CI: 1.80–6.69), LGT (OR = 2.71, 95%CI: 1.04–7.17), ZQFTN (OR = 2.30, 95%CI: 1.21–4.55). Then, ZQFTN yielded the better benefits for relieving the incidence of ADRs than GTW (OR = 0.68, 95%CI: 0.47–0.96).

Briefly, the SUCRA results showed that YXB (91.59%) was seemed to hold greater potential for relieving the incidence of ADRs among different CPMs, followed by WB (91.05%) and BQ (73.58%) (Fig. 4b, Table 3).

Meanwhile, based on summarizing the information about ADRs, the results also manifested that only 3 RCTs reported there was no RCTs during the clinical treatment process of both groups. Besides, the included trials involved 1421 cases of ADRs among patients with RA, and 601 cases occurred in CPMs groups, the remaining 820 subjects with ADRs were found in CM group. The majority of ADRs was transient and could remit spontaneously in a period of time or disappeared after reducing dosage, discontinuing administration and symptomatic treatment. Notably, there were 18 participants dropped out due to intolerance ADRs. The most common ADRs reported in the included trials were gastrointestinal reactions, namely nausea, vomiting, abdominal pain, anorexia and diarrhea; allergic reactions including rash with itching; other symptoms involving headache, dizziness, liver dysfunction and leucopenia. Herein the main ADRs for GTW were reproductive toxicity, such as menstrual disorder and oligospermatism. The skin rash accompanied by pruritus was frequent ADRs of ZQFTN.

### Cluster analysis

**Fig. 5:**
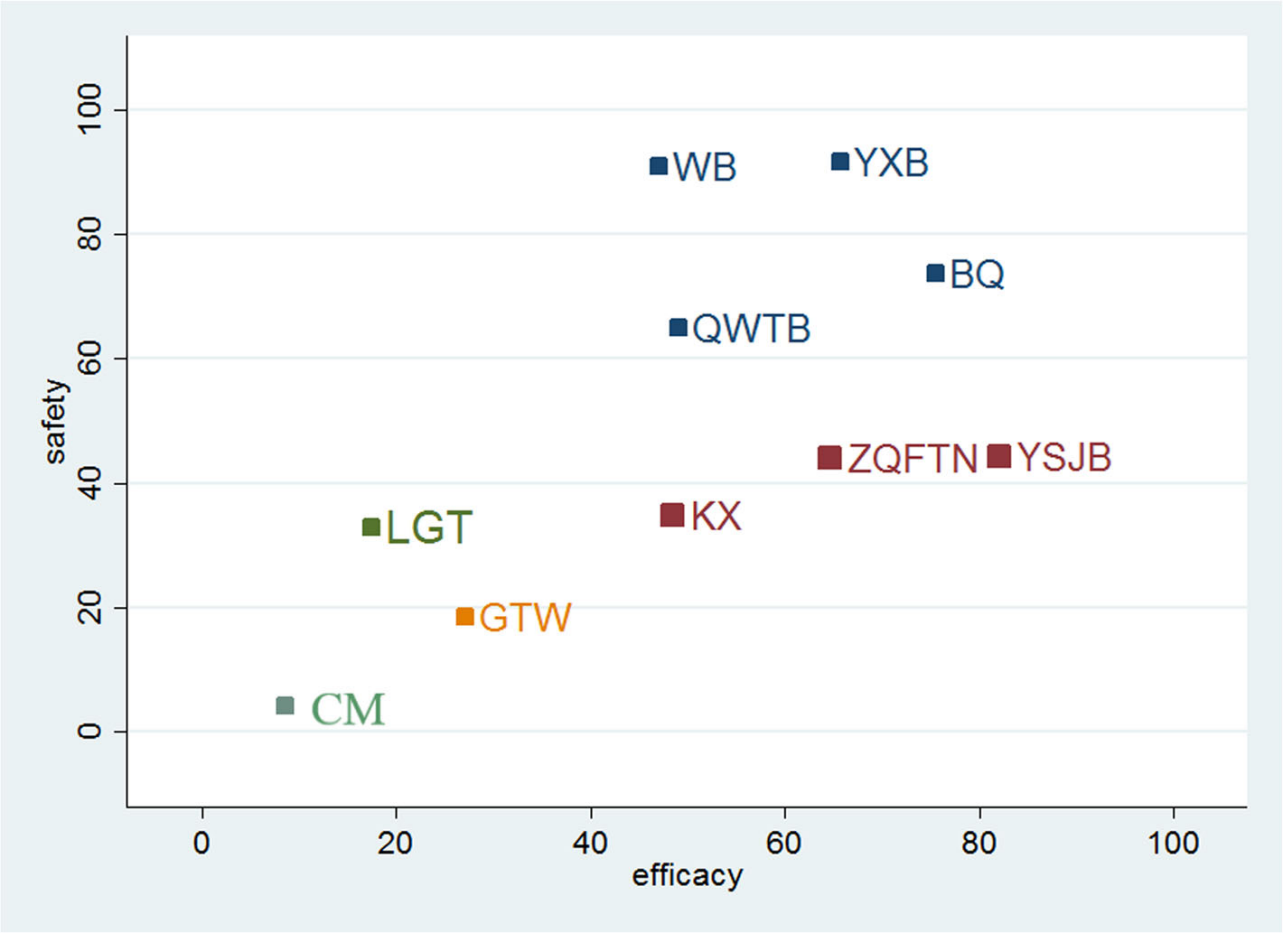
Cluster analysis plot of efficacy and safety. Note: the clinical effectiveness rate (X-axis) and the incidence of ADRs (Y-axis)

The cluster analysis was preformed to identify the promising therapeutic strategies from the different oral CPMs that concerning the clinical effectiveness rate and the incidence of ADRs simultaneously. As shown in Fig. 5, the results of the cluster analysis revealed that BQ, YXB, WB, and Qiweitongbi oral liquid (QWTB) were associated with a favorable benefits both in improving the clinical effectiveness rate and reducing the incidence of ADRs compared with the other CPMs. In contrast, CM single was the worst treatment strategy among the interventions considering the comprehensive rank of cluster analysis.

### Comprehensive investigation

**Fig. 6:**
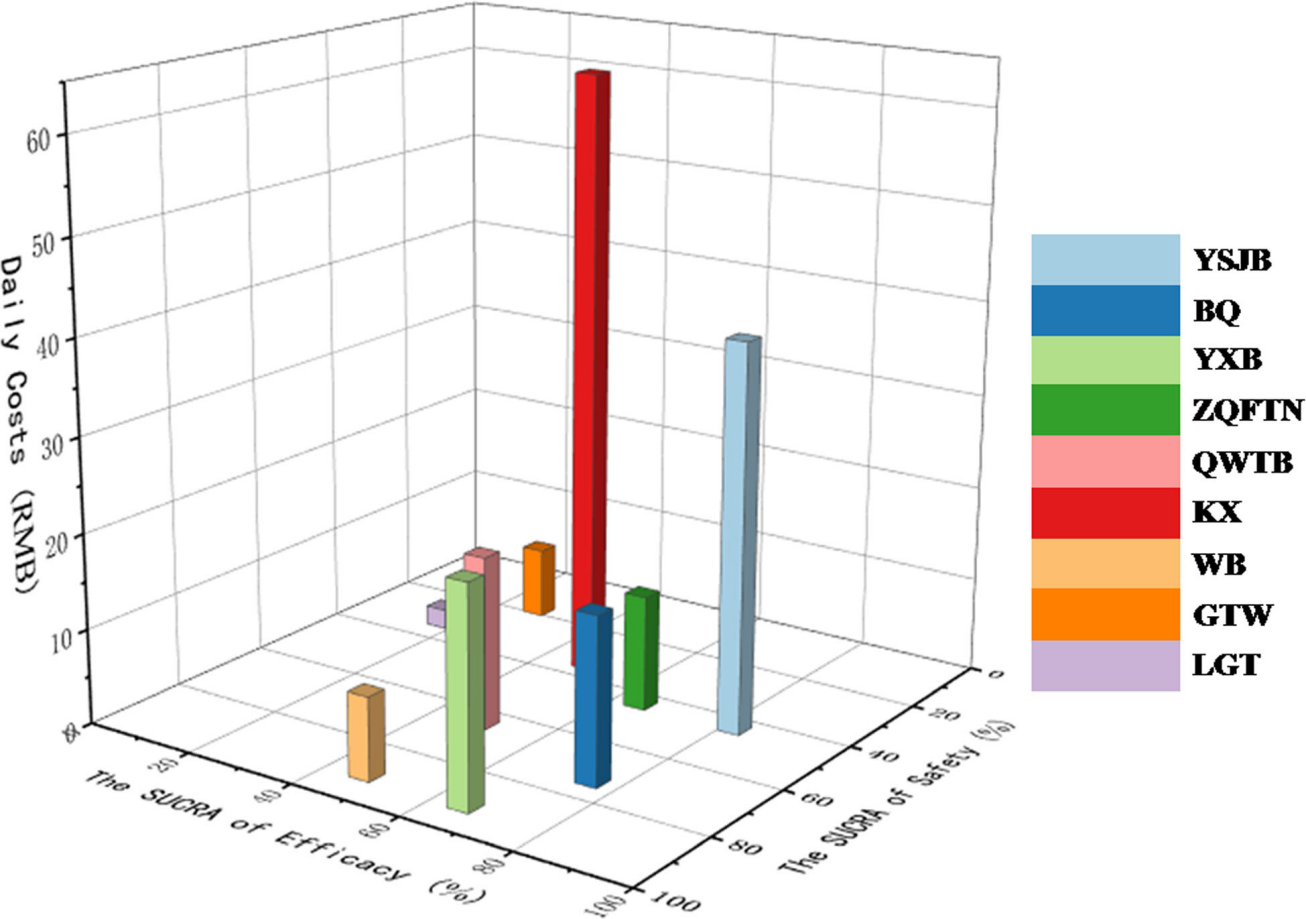
Comprehensive investigation for the comparative efficacy, safety and cost of CPMs

Comprehensive investigation regarding the comparative efficacy, safety and cost of oral CPMs for RA was conducted to guide decision-making, based on the specific information presented in Table 3, the results corroborated that BQ and YXB might be an attractive option in terms of the preferable improvement for efficacy and safety, and acceptable cost for patients with RA (Fig. 6). Nevertheless, although GTW and LGT did not possess the higher probability of both improving efficacy and safety, according to their daily cost, they were likely to be the least expensive choice.

### Publication bias and consistency test

**Fig. 7:**
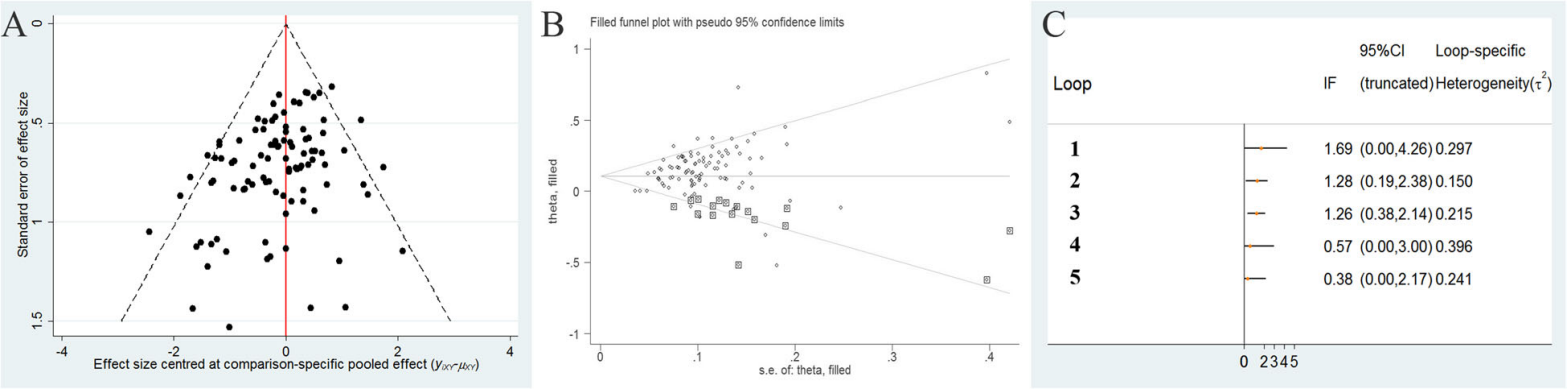
Funnel plot (**a**), Egger’s tests (**b**) and consistency test (**c**) of the clinical effectiveness rate. Note: 1: GTW-CM-YSJB; 2: BQ-CM-ZQFTN; 3: GTW-CM-ZQFTN; 4: FufangFSN-GTW-CM; 5: BQ-LGT-CM

As illustrated in Fig. 7a and Fig. 7b, publication bias of included RCTs for the clinical effectiveness rate was measured by funnel plots and Egger’s tests. According to Egger test (t = 3.41, *P* = .551 > .05) and the funnel plot after patched estimated the actual value of the combined effect, and the number of RCTs increased while no qualitative change produced, indicating that no obvious publication bias and small-size effects in our research.

The consistency test was also preformed for the clinical effectiveness rate (Fig. 7c), the inconsistency plot consisted of 4 triangular loops. The 95% CI of IF values was truncated at zero for 2 closed loops, indicating there is no evidence of significant inconsistency. Nevertheless, the significant inconsistency was observed in the loop of (BQ-CM-ZQFTN) (IF = 1.28, 95%CI = 0.19–2.38), and (GTW-CM-ZQFTN) (IF = 1.26, 95%CI = 0.38–2.14). Collectively, the results indicated some inconsistency in this loop.

### The secondary outcomes

**Fig. 8:**
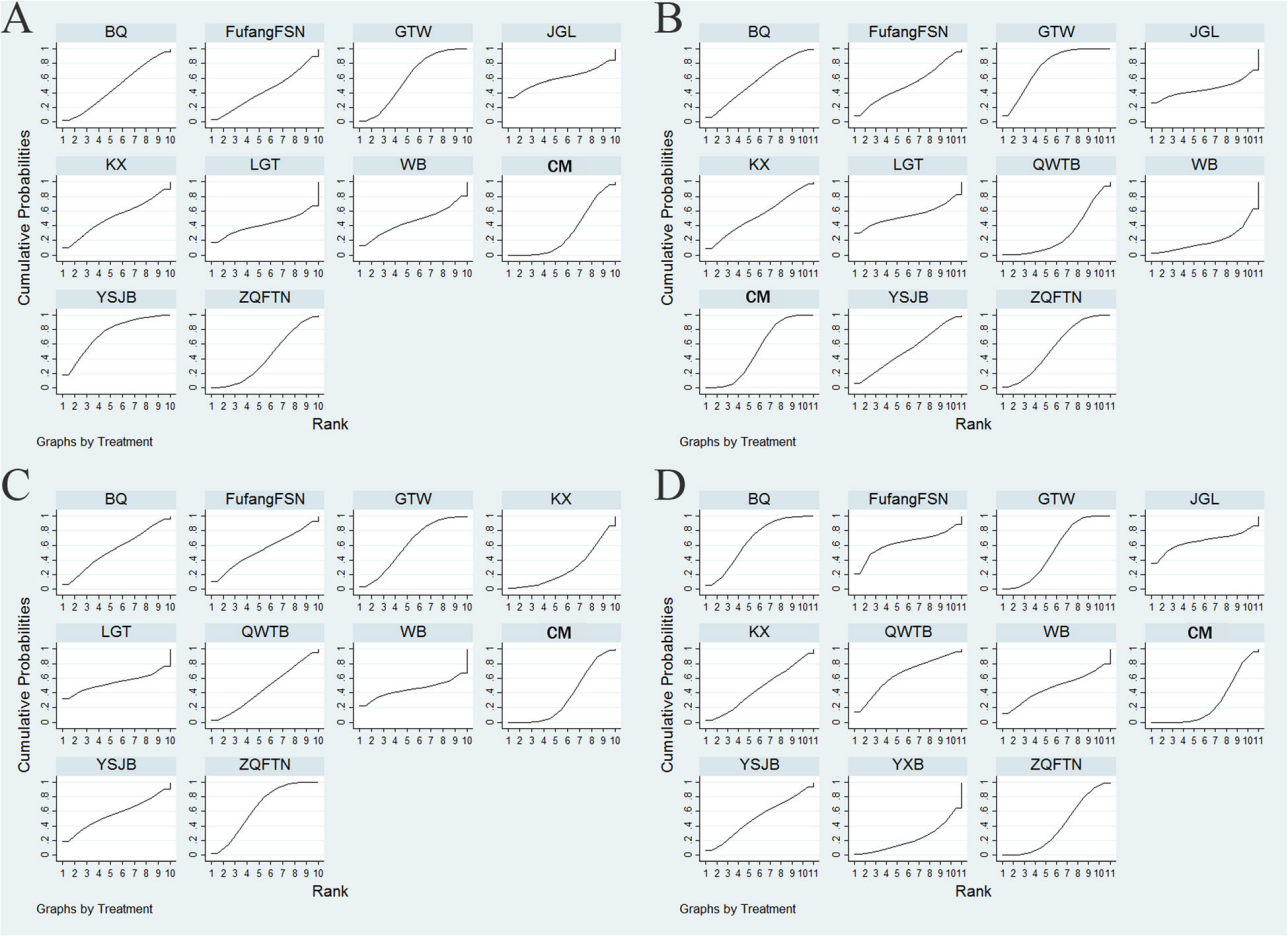
Rank of the cumulative probabilities for secondary outcomes. Note: **a**: joint tenderness; **b**: joint swelling; **c**: morning stiffness; **d**: ESR

The secondary outcomes of present NMA included joint tenderness (49 RCTs), joint swelling (52 RCTs), morning stiffness (62 RCTs), and ESR (78 RCTs). Disappointingly, the results revealed that no significant difference was found among these comparisons under these outcomes (**Supplementary Table** **3** and **Supplementary Table** **4**). With respect to the joint tenderness, YSJB (74.7%) exhibited the greatest possibility with improving the joint tenderness over other CPMs, followed by GTW (59.8%) and JGL (59.8%), Fig. 8a. GTW had 75.5% probability of being ranked as the most effective treatment for modulating joint swelling for patients with RA, followed by BQ (57.7%), ZQFTN (55.8%), Fig. 8b. In terms of morning stiffness, ZGFTN (64.9%) the highest probability of providing symptomatic benefits for morning stiffness, followed by GTW (60.9%) and YSJB (56.4%), Fig. 8c. Moreover, BQ (67.0%) had the greatest possibility of achieving a considerable improvement in ESR, followed by QWTB (65.4%) and JGL (65.3%), Fig. 8d. In general, GTW could confer favourable response with higher SUCRA values on improving joint tenderness, joint swelling, and morning stiffness. Additionally, the SUCRA values of each CPM for secondary outcomes were presented in Table 4.

**Table 4:** The SUCRA values of included CPMs for secondary outcomes

CPMs	joint tenderness	joint swelling	morning stiffnes	ESR
Biqi capsule	48.4%	**57.7%**	54.3%	**67.0%**
Fufang-Fengshining capsule	43.3%	51.9%	54.2%	62.9%
Jingulian capsule	**59.8%**	45.7%	–	**65.3%**
Kunxian capsule	52.0%	54.7%	28.6%	46.1%
glucosides of Tripterygium Wilfordii tablet	**59.8%**	**75.5%**	**60.9%**	54.2%
Leigongteng tablet	41.8%	54.4%	54.2%	–
Qiweitongbi oral liquid	–	29.0%	46.5%	**65.4%**
Wangbi tablet	46.2%	20.6%	44.8%	47.8%
Yishenjuanbi pill	**74.7%**	52.7%	**56.4%**	51.5%
Yuxuebi capsule	–	–	–	22.3%
Zhengqingfengtongning tablet	42.2%	**55.0%**	**64.9%**	39.7%

Note: Results in bold possessed the higher SUCRA values

## Discussion

Since there were various CPMs against RA about which there was no consensus yet, the present NMA was conducted to generate hierarchy of treatment rankings, the ranking probabilities for each CPM were further calculated with regard to their comparative efficacy, safety, and cost under different endpoints to provide basis for selecting alternatives and establish the optimal choices. According to the results of comprehensive synthesis, BQ and YXB probably had a favorable balance in consideration of benefits, tolerability and daily cost. Furthermore, as the least expensive choice, GTW was associated with displaying a trend of relieving joint tenderness, joint swelling, and morning stiffness for patients with RA. Remarkably, the choice of specific CPMs should make comprehensive consideration of the various factors simultaneously, for example, the high-quality evidence-based research, the conditions and state of patients, clinical practice and experience of physicians, the policy of insurance companies or the marketing efforts of pharmaceutical companies. More significantly, it is crucial that paying close attention to severe ADRs of CPMs during the long duration for treating RA in order to promote rational drug use and ensure medical safety.

Recently, BQ has been widely applied for the clinical treatment of RA owing to it can offer clear advantages for improving clinical symptoms, such as the soreness, swelling, stiffness of joint, and reducing inflammatory markers [41]. It is noteworthy that, there is growing evidence that its active ingredients may possibly contribute to both efficacy and toxicity of BQ [42]. On the one hand, cryptotanshinone, brucine and strychnine are the major anti-inflammatory components to induce reactive oxygen species-mediated apoptosis, regulate cytokine expression, suppress the proliferation of synovial fibroblasts and inhibits the secretion of nitric oxide, NO synthase, and interleukin (IL)-6 [43–45]. On the other hand, the renal injury caused by strychnine and brucine was reported in many clinical cases, however, it is also proved that Radix et Rhizoma Glycyrrhizae in BQ can slow the absorption of these alkaloids to protect the condition of kidney [46, 47]. Also, there are concern regarding that their biological effects are dose-dependent; hence it is required to reduce the toxicity of the seeds through processing, compatibility, clinical monitoring, and dose reduction [48]. Similar to other efficacious TCM formulations, YXB and WB have been administered for managing and treating RA in China for decades, it has been demonstrated that YXB and WB could exert notable therapeutic effects on regulating immune responses, ameliorating joint destruction and relieving pain for patients with RA [49, 50]. The critical mechanism is probably related to inhibit the NF-κB and JAK-STAT3 signaling pathways on adjuvant-induced arthritis rats [51]. As traditional medicines have a rich resource of bioactive components, GTW is a natural drug product components which derived from Radix et Rhizoma Tripterygii Wilfordii, a well-known irreplaceable immunosuppressant against inflammation, pain, and immune regulation for centuries in China, accompanying with the unavoidable reproductive toxicity and hepatotoxicity [52, 53]. It is well documented that on behalf of diterpenoid constituents, triptolide and triptophenolide exhibit impressive and remarkable anti-inflammatory, immunosuppressive, and antifertility activities [54, 55]. Alternatively, ZQFTN is isolated from Caulis Sinomenii with long medication history in China over thousands of years, has great application values for treating RA in virtue of various pharmacological and biological effects including analgesia, anti-inflammatory properties, and immune suppression [56, 57]. Briefly, the recent advancements in pharmacological research on the field of CPMs for treating RA support our evidence-base medicine findings.

To the best of our knowledge, this is the first systematic review with Bayesian NMA that comprehensive investigated and evaluated the different oral CPMs in the treatment of RA, and recommended a rank order based on efficacy, safety, and cost. Admittedly, our research provides the available evidence for clinicians who is puzzled by some inevitable problems involving lack of comparative efficacy data, drug toxicity, high cost for application of the current therapeutic strategies among CPMs. First, through the comprehensive and systematic literature search and subsequently reproducible eligibility criteria, ultimately, 10,213 patients with RA from 37 RCTs involving 11 oral CPMs were enrolled and evaluated in present NMA. With regard to the selected oral CPMs, we focused on those were listed and recommended in *the Catalogue of Drugs for Basic National Medical Insurance* (The 2017 Edition) by the National Medical Products Administration in China [25], with the aim of offering a comprehensive overview for TCM, especially the modern preparation such as CPMs treating RA. Second, our research not only concerned about the efficacy outcomes including clinical effectiveness rate, joint tenderness, joint swelling, morning stiffness, and ESR, but also paid attention to the incidence of ADRs and daily cost. Apart from above merits, the hierarchy was calculated based on the SUCRA to identify the optimal treatment for each outcome; the cluster analysis and comprehensive investigation were preformed to estimate the superior CPMs accounting for both efficacy, safety and cost. Lastly, the quality assessment was preformed for the enrolled RCTs, the comparison-adjusted funnel plot, and Egger’s test were adopt to measure publication bias and small-size effects, and the consistency test in node-splitting analysis for each closed loop was conducted to explore the credibility of both direct and indirect evidence, these methodological analysis can enhance reliability and accuracy of our findings.

Despite the present findings help to fill the gap created by the lack of head-to-head comparisons of different oral CPMs in people with RA, this research also had some limitations as below. First, the majority of the selected RCTs had uncertain or high risk of bias in the domain of allocation concealment and blinding methods. Besides, the sample size of some eligible trials was relatively small; these factors might contribute to the exaggerated therapeutic effects of treatment and prevent stronger conclusions. Second, some raw data about long-term endpoint, safety profile, disease status, and patients’ condition was insufficient to support further follow-up analysis and subgroup analysis for different disease courses and syndrome patterns, hence, suggesting that the clinical trials of patients with RA should illustrate and provide more evidence about long-term efficacy, treatment details, and disease information. Meanwhile, there was some inconsistency across direct and indirect comparisons; more head-to-head RCTs for different oral CPMs and commonly known western medications are warranted to draw more robust and reliable conclusions. Moreover, the recruited patients with RA in present NMA were Asian descent; accordingly, the results of our NMA should be interpreted with caution for non-Asian population.

## Conclusion

Overall, the current evidence suggests that BQ, YXB and GTW are associated with the most preferable, beneficial and cost-effective options for patients with RA in terms of efficacy, safety and cost, although additional results from multi-center trials and high-quality studies will be pivotal for confirming and supporting our findings.

## Supplementary information

**Additional file 1 Supplementary file 1.** PRISMA check list. **Supplementary file 2.** Search strategy. **Supplementary file 3.** PRISMA flow chart. **Supplementary Table S1.** The product information (raw materials, labeled efficacy, indications) of CPMs. **Supplementary Table S2.** The baseline characteristics of the included RCTs and subjects. **Supplementary Table S3.** Results of the network meta-analysis for joint tenderness (upper-right quadrant) and the joint swelling (lower-left quadrant). **Supplementary Table S4.** Results of the network meta-analysis for morning stiffness (upper-right quadrant) and ESR (lower-left quadrant). **Supplementary Table S5.** Risk-of-bias judgements for the included RCTs (RoB 2)

## Data Availability

Specific study data are available from the authors on request.

## Abbreviations

ACR: The American College of Rheumatology
ADRs: Adverse drug reactions
BQ: Biqi capsule
CBMdisc: The China Biology Medicine disc
CI: Credible intervals
CMs: Conventional medicines
CNKI: The China National Knowledge Infrastructure Database
CPMs: Chinese patent medicines
DMARDs: Disease-modifying anti-rheumatic drugs
ESR: Erythrocyte sedimentation rate
FufangFSN: Fufang-Fengshining capsule/tablet
GTW: glucosides of Tripterygium Wilfordii tablet
IF: Inconsistency factors
IL: Interleukin
JGL: Jingulian capsule
KX: Kunxian capsule
LGT: Leigongteng tablet
MCMC: The Marko chain Monte Carlo
MD: Mean differences
NMA: Network meta-analysis
NSAIDs: Nonsteroidal anti-inflammatory drugs
OR: Odds ratios
PICOS: Patients, intervention, comparison, outcome, and study design
PRISMA: The Preferred Reporting Items for Systematic reviews and Meta-Analyses
QWTB: Qiweitongbi oral liquid
RA: Rheumatoid arthritis
RCTs: Randomized controlled trials
SUCRA: Surface under the cumulative ranking curve
TCM: Traditional Chinese medicine
VIP: The Cqvip Database
WB: Wangbi tablet
YSJB: Yishenjuanbi pill
YXB: Yuxuebi capsule/tablet
ZQFTN: Zhengqingfengtongning tablet/sustained-release tablet/capsule
