# Supplementary material for: Comparative efficacy, safety and cost of oral Chinese patent medicines for rheumatoid arthritis: a Bayesian network meta-analysis
Source: BMC Complement Med Ther. 2020 Jul 6;20:210. doi: 10.1186/s12906-020-03004-4 (PMC7339567; doi:10.1186/s12906-020-03004-4)
Supplement: Supplementary file 1 — Additional file 1 Supplementary file 1. PRISMA check list. Supplementary file 2. Search strategy. Supplementary file 3. PRISMA flow chart. Supplementary Table S1. The product information (raw materials, labeled efficacy, indications) of CPMs. Supplementary Table S2. The baseline characteristics of the included RCTs and subjects. Supplementary Table S3. Results of the network meta-analysis for joint tenderness (upper-right quadrant) and the joint swelling (lower-left quadrant). Supplementary Table S4. Results of the network meta-analysis for morning stiffness (upper-right quadrant) and ESR (lower-left quadrant). Supplementary Table S5. Risk-of-bias judgements for the included RCTs (RoB 2) [file 12906_2020_3004_MOESM1_ESM.docx]

**Supplementary Material**

**Comparative efficacy, safety and cost of oral Chinese patent medicines for rheumatoid arthritis: a Bayesian network meta-analysis**

**File S1.** PRISMA check list

| **Section/topic** | **#** | **Checklist item** | **Reported on page #** |
| --- | --- | --- | --- |
| **TITLE** | | |  |
| Title | 1 | Identify the report as a systematic review incorporating a network meta-analysis (or related form of meta-analysis). | 1 |
| **ABSTRACT** | | |  |
| Structured summary | 2 | Provide a structured summary including, as applicable:  Background: main objectives  Methods: data sources; study eligibility criteria, participants, and interventions; study appraisal; and synthesis methods, such as network meta-analysis.  Results: number of studies and participants identified; summary estimates with corresponding confidence/credible intervals; treatment rankings may also be discussed. Authors may choose to summarize pairwise comparisons against a chosen treatment included in their analyses for brevity.  Discussion/Conclusions: limitations; conclusions and implications of findings.  Other: primary source of funding; systematic review registration number with registry name. | 1 |
| **INTRODUCTION** | | |  |
| Rationale | 3 | Describe the rationale for the review in the context of what is already known, including mention of why a network meta-analysis has been conducted | 2 |
| Objectives | 4 | Provide an explicit statement of questions being addressed with reference to participants, interventions, comparisons, outcomes, and study design (PICOS). | 2 |
| **METHODS** | | |  |
| Protocol and registration | 5 | Indicate if a review protocol exists and if and where it can be accessed (e.g., Web address), and, if available, provide registration information including registration number. | / |
| Eligibility criteria | 6 | Specify study characteristics (e.g., PICOS, length of follow-up) and report characteristics (e.g., years considered, language, publication status) used as criteria for eligibility, giving rationale. Clearly describe eligible treatments included in the treatment network, and note whether any have been clustered or merged into the same node (with justification). | 3-4 |
| Information sources | 7 | Describe all information sources (e.g., databases with dates of coverage, contact with study authors to identify additional studies) in the search and date last searched. | 3 |
| Search | 8 | Present full electronic search strategy for at least one database, including any limits used, such that it could be repeated. | 3 |
| Study selection | 9 | State the process for selecting studies (i.e., screening, eligibility, included in systematic review, and, if applicable, included in the meta-analysis). | 3-4 |
| Data collection process | 10 | Describe method of data extraction from reports (e.g., piloted forms, independently, in duplicate) and any processes for obtaining and confirming data from investigators. | 4 |
| Data items | 11 | List and define all variables for which data were sought (e.g., PICOS, funding sources) and any assumptions and simplifications made. | 4 |
| Geometry of the network | 12 | Describe methods used to explore the geometry of the treatment network under study and potential biases related to it. This should include how the evidence base has been graphically summarized for presentation, and what characteristics were compiled and used to describe the evidence base to readers | 5 |
| Risk of bias within individual studies | 13 | Describe methods used for assessing risk of bias of individual studies (including specification of whether this was done at the study or outcome level), and how this information is to be used in any data synthesis. | 4 |
| Summary measures | 14 | State the principal summary measures (e.g., risk ratio, difference in means). Also describe the use of additional summary measures assessed, such as treatment rankings and surface under the cumulative ranking curve (SUCRA) values, as well as modified approaches used to present summary findings from meta-analyses. | 4-5 |
| Planned methods of analysis | 15 | Describe the methods of handling data and combining results of studies for each network meta-analysis. This should include, but not be limited to: Handling of multigroup trials; Selection of variance structure; Selection of prior distributions in Bayesian analyses; and Assessment of model fit. | 4-5 |
| Assessment of inconsistency | 16 | Describe the statistical methods used to evaluate the agreement of direct and indirect evidence in the treatment network(s) studied. Describe efforts taken to address its presence when found. | 5 |
| Risk of bias across studies | 17 | Specify any assessment of risk of bias that may affect the cumulative evidence (e.g., publication bias, selective reporting within studies) | 5 |
| Additional analyses | 18 | Describe methods of additional analyses if done, indicating which were prespecified. This may include, but not be limited to, the following: Sensitivity or subgroup analyses; Meta-regression analyses; Alternative formulations of the treatment network; and Use of alternative prior distributions for Bayesian analyses (if applicable). | 5 |
| **RESULTS** | | |  |
| Study selection | 19 | Give numbers of studies screened, assessed for eligibility, and included in the review, with reasons for exclusions at each stage, ideally with a flow diagram. | 5-6 |
| Presentation of network structure | 20 | Provide a network graph of the included studies to enable visualization of the geometry of the treatment network. | 5-6 |
| Summary of network geometry | 21 | Provide a brief overview of characteristics of the treatment network. This may include commentary on the abundance of trials and randomized patients for the different interventions and pairwise comparisons in the network, gaps of evidence in the treatment network, and potential biases reflected by the network structure. | 6 |
| Study characteristics | 22 | For each study, present characteristics for which data were extracted (e.g., study size, PICOS, follow-up period) and provide the citations. | 5-6 |
| Risk of bias within studies | 23 | Present data on risk of bias of each study and, if available, any outcome level assessment. | 6 |
| Results of individual studies | 24 | For all outcomes considered (benefits or harms), present, for each study: 1) simple summary data for each intervention group, and 2) effect estimates and confidence intervals. Modified approaches may be needed to deal with information from larger networks. | 6-9 |
| Synthesis of results | 25 | Present results of each meta-analysis done, including confidence/credible intervals. In larger networks, authors may focus on comparisons versus a particular comparator (e.g., placebo or standard care), with full findings presented in an appendix. League tables and forest plots may be considered to summarize pairwise comparisons. If additional summary measures were explored (such as treatment rankings), these should also be presented. | 6-9 |
| Exploration for inconsistency | 26 | Describe results from investigations of inconsistency. This may include such information as measures of model fit to compare consistency and inconsistency models, P values from statistical tests, or summary of inconsistency estimates from different parts of the treatment network. | 8-9 |
| Risk of bias across studies | 27 | Present results of any assessment of risk of bias across studies for the evidence base being studied. | 8-9 |
| Results of additional analyses | 28 | Give results of additional analyses, if done (e.g., sensitivity or subgroup analyses, meta-regression  analyses, alternative network geometries studied, alternative choice of prior distributions for  Bayesian analyses, and so forth). | 8-9 |
| **DISCUSSION** | | |  |
| Summary of evidence | 29 | Summarize the main findings, including the strength of evidence for each main outcome; consider their relevance to key groups (e.g., health care providers, researchers, and policymakers). | 9-11 |
| Limitations | 30 | Discuss limitations at study and outcome level (e.g., risk of bias), and at review level (e.g., incomplete retrieval of identified research, reporting bias). Comment on the validity of the assumptions, such as transitivity and consistency. Comment on any concerns regarding network geometry (e.g., avoidance of certain comparisons). | 11-12 |
| Conclusions | 31 | Provide a general interpretation of the results in the context of other evidence, and implications for future research. | 12 |
| **FUNDING** | | |  |
| Funding | 32 | Describe sources of funding for the systematic review and other support (e.g., supply of data); role of funders for the systematic review. This should also include information regarding whether funding has been received from manufacturers of treatments in the network and/or whether some of the authors are content experts with professional conflicts of interest that could affect use of treatments in the network. | 13 |

**File S2.** Search strategy.

**Search strategy of PubMed**

1. Rheumatoid Arthritis[MeSH Terms]
2. Rheumatoid[Title/Abstract]
3. Arthritis[Title/Abstract]
4. Rheumatoid Nodule[Title/Abstract]
5. #1 OR #2 OR #3 OR #4
6. Fengshigutong[Title/Abstract]
7. Zhuifengtougu[Title/Abstract]
8. Fengshiqutong[Title/Abstract]
9. Fuguigutong[Title/Abstract]
10. Fufangxuelian[Title/Abstract]
11. Hanshibi[Title/Abstract]
12. Jinwugutong[Title/Abstract]
13. Mugua[Title/Abstract]
14. Qiweitongbi[Title/Abstract]
15. Wantongjingu[Title/Abstract]
16. Dangguiniantong[Title/Abstract]
17. Ermiao[Title/Abstract]
18. Huamoyan[Title/Abstract]
19. Shirebi[Title/Abstract]
20. Simiao[Title/Abstract]
21. Tongfengding[Title/Abstract]
22. Tongfengshu[Title/Abstract]
23. Zhengqingfengtongning[Title/Abstract]
24. Fufangfengshining[Title/Abstract]
25. Fengshimaqian[Title/Abstract]
26. Guanjiekebi[Title/Abstract]
27. Heigutengzhuifenghuoluo[Title/Abstract]
28. Huli[Title/Abstract]
29. Jiaweitianma[Title/Abstract]
30. Jingulian[Title/Abstract]
31. Kanglangchuang[Title/Abstract]
32. Kunxian[Title/Abstract]
33. Shufengdingtong[Title/Abstract]
34. Shenyansiwei[Title/Abstract]
35. Malizhongzitiquwu[Title/Abstract]
36. Maizhiling[Title/Abstract]
37. Mailuoshutong[Title/Abstract]
38. Panlongqi[Title/Abstract]
39. Yuxuebi[Title/Abstract]
40. Fengshiye[Title/Abstract]
41. Pulean[Title/Abstract]
42. Wangbi[Title/Abstract]
43. Bixiefenqing[Title/Abstract]
44. Biqi[Title/Abstract]
45. Duhuojisheng[Title/Abstract]
46. Jintiange[Title/Abstract]
47. Shenkangning[Title/Abstract]
48. Tianmazhuanggu[Title/Abstract]
49. Tongbi[Title/Abstract]
50. Yishenjuanbi[Title/Abstract]
51. Zhuanggushenjin[Title/Abstract]
52. Zhuangyaojianshen[Title/Abstract]
53. Leigongteng[Title/Abstract]
54. Leigongtengduogan[Title/Abstract]
55. Leigongtengduodai[Title/Abstract]
56. Leigongtengjiasu[Title/Abstract]
57. Tripterygium[Title/Abstract]
58. Tripterygium glycosides[Title/Abstract]
59. Tripterygium polyglycosides[Title/Abstract]
60. glucosides of Tripterygium wilfordii[Title/Abstract]
61. Tripterygium glycosides tablet[Title/Abstract]
62. Tripterygium polyglycosides tablet[Title/Abstract]
63. Tripterygium tablet[Title/Abstract]
64. Triptolide[Title/Abstract]
65. TWP[Title/Abstract]
66. TwHF[Title/Abstract]
67. #6 OR #7 OR #8 OR #9 OR #10 OR#11 OR#12 OR #13 OR #14 OR #15 OR #16 OR #17 OR #18 OR #19 OR #20 OR #21 OR #22 OR #23 OR #24OR #25 OR #26 OR #27 OR #28 OR #29 OR #30 OR #31 OR #32 OR #33 OR #34 OR #35 OR #36 OR #37OR #38 OR #39 OR #40 OR #41 OR #42 OR #43 OR #44OR #45OR #46OR #47OR #48OR #49OR #50OR #51OR #52OR #53OR #54OR #55OR #56OR #57OR #58OR #59OR #60OR #61OR #62OR #63OR #64OR #65OR #66
68. randomized controlled trial[Publication Type]
69. controlled clinical trial[Publication Type]
70. randomized[Title/Abstract]
71. placebo[Title/Abstract]
72. randomly[Title/Abstract]
73. trial[Title/Abstract]
74. groups[Title/Abstract]
75. "drug therapy"[Subheading]
76. #68 OR #69 OR #70 OR #71 OR #72 OR #73 OR #74 OR #75
77. animals[MeSH Terms]
78. humans[MeSH Terms]
79. #77 NOT #78
80. #76 NOT #79
81. #5 AND #67 AND #80

**File S3.** PRISMA flow chart.


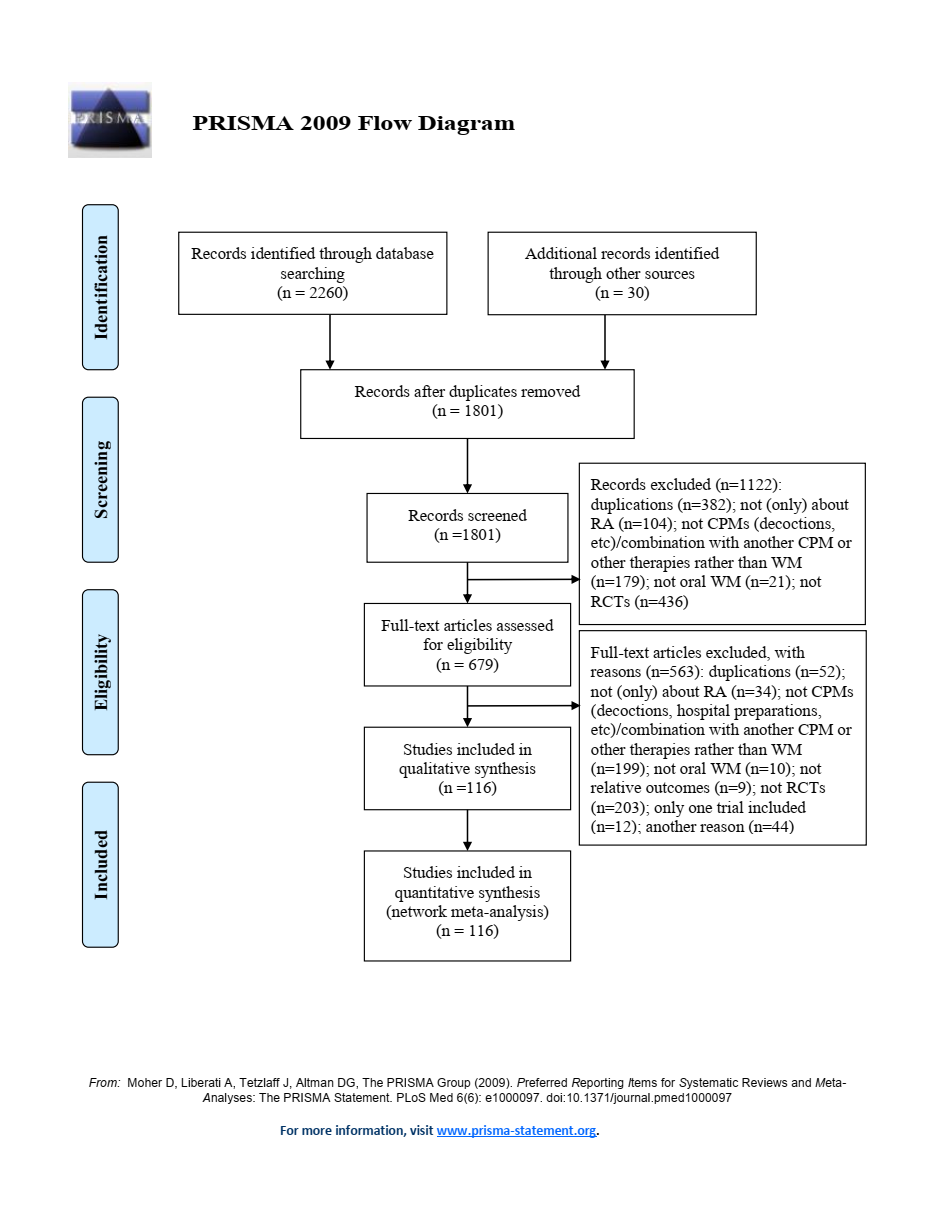


**Table S1.** The product information (raw materials, labeled efficacy, indications) of CPMs.

| CPM name | Raw materials | Labeled efficacy | Indications |
| --- | --- | --- | --- |
| Biqi capsule | Strychni Semen Pulveratum, Pheretima, Codonopsis Radix, Poria, Atractylodis Macrocephalae Rhizoma, Chuanxiong Rhizoma, Salviae Miltiorrhizae Radix et Rhizoma, Notoginseng Radix et Rhizoma, Achyranthis Bidentatae Radix, Glycyrrhizae Radix et Rhizoma | Invigorating Qi and nourishing blood, dispelling wind and eliminating dampness, activating blood and relieving pain. | Insufficiency of vital energy and blood, rheumatic stasis, muscle and joint soreness, joint swelling, stiffness and deformation, muscular atrophy; rheumatism, rheumatoid arthritis, lumbar muscle degeneration, and soft tissue injury that belong to the above syndromes |
| Fufang-Fengshining capsule/ tablet | Zanthoxyli Radix, Stauntoniae Cauliset Folium, Caulis Tinosporae Sinensis, Caulis Entadae Phaseoloidis, Clematidis Radix et Rhizoma, Crotonis Crassifolii Radix | Dispelling wind and eliminating dampness, activating blood and dispersing stasis, relieving tendons and alleviating pain. | Rheumatism arthralgia |
| Jingulian capsule | Herba Gaultheriae, Ramulus et Folium Schefflerea Leucanthae, Sargentodoxae Caulis, Radix Alangii, Psammosilenes Radix | Dispelling wind and eliminating dampness, reducing swelling and alleviating pain. | Joint swelling and pain, difficulty in extension and flexion caused by rheumatism obstruction. |
| Kunxian capsule | Radix Tripterygii Hypoglauci, Epimedii Folium, Lycii Fructus, Cuscutae Semen | Tonifying kidney and activating collaterals, dispelling wind and eliminating dampness. | Rheumatoid arthritis belong to rheumatic stasis with kidney deficiency syndromes, joint swelling and pain, difficulty in extension and flexion, morning stiffness, joint tenderness, joint chills, soreness and weakness of waist and knees, light tongue texture, white tongue moss, deep and thin pulse. |
| glucosides of Tripterygium Wilfordii tablet | Glucosides of Tripterygium Wilfordii | Dispelling wind and detoxicating, eliminating dampness and reducing swelling, relaxing tendons and removing obstruction. It has anti-inflammatory and inhibition effect on cellular immunity and humoral immunity. | Rheumatoid arthritis caused by wind-dampness and heat-blood stasis syndrome, toxicity and pathogenic blockade, nephrotic syndrome, Bechet's disease, lepra reaction, autoimmune hepatitis |
| Leigongteng tablet | The extraction of Tripterygium Wilfordii | It has the anti-inflammatory and immunosuppressive functions. | Rheumatoid arthritis |
| Qiweitongbi oral liquid | Formica fusca Linnaeus, Sinomenii Caulis, Spatholobi Caulis, Pyrolae Herba, Ramulus Photiniae, Homalomenae Rhizoma, Clematidis Radix et Rhizoma | Tonifying kidney and strengthening bones, expelling wind and eliminating obstruction. | Syndrome belongs to insufficiency of liver and kidney, rheumatism obstructs collaterals. Joint swelling and pain, difficulty in extension and flexion, soreness and weakness of waist and knees, scleroma, morning stiffness, moved with difficulty, light or dark tongue texture, white and thin tongue moss. |
| Wangbi tablet | Rehmanniae Radix, Rehmanniae Radix Praeparata, Dipsaci Radix, Aconiti Lateralis Radix Praeparata, Angelicae Pubescentis Radix, Drynariae Rhizoma, Cinnamomi Ramulus, Epimedii Folium, Saposhnikoviae Radix, Clematidis Radix et Rhizoma, Gleditsiae Spina, goat bone, Paeoniae Radix Alba, Cibotii Rhizoma, Anemarrhenae Rhizoma, Lycopodii Herba, Carthami Flos | Nourishing liver and kidney, strengthening bones and muscles, dispelling wind-damp, dredging meridians. | Rheumatoid arthritis caused by liver and kidney insufficiency and rheumatism obstructing collaterals, Muscle and joint pain, focal swelling, stiffness, deformity, difficulty in extension and flexion, soreness and weakness of waist and knees, intolerance to cold, fatigue, etc.. |
| Yishenjuanbi pill | Drynariae Rhizoma, Rehmanniae Radix Praeparata, Angelicae Sinensis Radix, Cynanchi Paniculati Radix et Rhizoma, Eupolyphaga Steleophaga, Bombyx Batryticatus, Scolopendra, Scorpio, Vespae Nidus, Pheretima, Zaocys, Corydalis Rhizoma, Pyrolae Herba, Epimedii Folium, Rhizoma seu Herba Aristolochiae Mollissimae, Erodii Herba Geranii Herba, Spatholobi Caulis, Humulus Scandens (Lour.) Merr., Rehmanniae Radix, PolygoniCuspidatiRhizomaet Radix | Warming and recuperating kidney yang, supplementing kidney and strengthening du meridian, dispelling wind and eliminating pathogenic factors. | Obstinate arthralgia (rheumatoid arthritis) included joint pain, swelling, red, hot and pain, difficulty in extension and flexion, muscular pain, gaunt, stiff, or malformation. |
| Yuxuebi capsule/ tablet | Olibanum, Myrrha, Carthami Flos, Clematidis Radix et Rhizoma, Cyathulae Radix, Cyperi Rhizoma, Curcumae Longae Rhizoma, Angelicae Sinensis Radix, Salviae Miltiorrhizae Radix et Rhizoma, Chuanxiong Rhizoma, Astragali Radix Praeparata Cum Melle | Promoting blood circulation and removing blood stasis, dredging collaterals and relieving pain. | *Bi* disease caused by blood stasis in collaterals, acute pain of muscles and joints, refused to press, fixed, rigid joints or ecchymosis |
| Zhengqingfengtongning tablet/ sustained-release tablet/ capsule | Sinomenine | Dispelling wind and eliminating dampness, promoting blood circulation and dredging collaterals, reducing swelling and alleviating pain. | *Bi* syndrome induced by wind, cold and dampness, muscle soreness, joint pain and swelling, difficulty in extension and flexion, stiffness, numbness of the limbs; rheumatoid arthritis and rheumatoid arthritis that belong to the above syndromes |

**Table S2.** The baseline characteristics and reference list of the included RCTs.

**The baseline characteristics of the included RCTs**

| Study ID | Gender(M/F) | Age(range) | Sample size (I/C) | Intervention (CPMs group) | Comparison (WM group) | Course | Outcomes |
| --- | --- | --- | --- | --- | --- | --- | --- |
| Liu W2007^[1]^ | 30/10 | 16-65 | 30/10 | BQ1.2g tid | ZQFTN80mg tid | 12w | ①②③④⑤⑥ |
| Bai RX2016^[2]^ | 47/189 | / | 178/58 | BQ1.2g tid | ZQFTN80mg tid | 16w | ①②⑥ |
| Liu P2012^[3]^ | 28/62 | 44-65 | 48/42 | BQ1.2g bid/tid | LGT66μg tid | 12w | ①③④⑤ |
| Jie HY2012^[4]^ | 13/77 | 19-49 | 44/46 | BQ1.2g tid | MTX10mg qw | 12w | ①③④⑤⑥ |
| Jia Q2018^[5]^ | 47/33 | 22-52 | 40/40 | BQ1.2g tid+MTX7.5mg qw | MTX7.5mg qw | 12w | ①② |
| Wan HJ2012^[6]^ | 33/19 | / | 26/26 | BQ1.2g tid+MTX7.5mg qw | MTX7.5mg qw | 12w | ①②③④ |
| Dai LP2016^[7]^ | 47/49 | 32-79 | 48/48 | BQ1.2g tid | loxoprofen sodium60mg tid | 8w | ①② |
| Liu W2006^[8]^ | 29/113 | 20-65 | 71/71 | BQ1.2g tid | nimesulide100mg bid | 8w | ①②③④⑤⑥ |
| Hu XM2006^[9]^ | 13/47 | 23-64 | 30/30 | BQ1.2g tid | ZQFTN60mg tid | 12w | ①②③④⑤⑥ |
| Bai HT2009^[10]^ | 16/52 | 21-76 | 50/18 | BQ1.2g tid | MTX10mg qw | 8w | ①②⑤⑥ |
| Yu WH2017^[11]^ | 102/114 | 24-54 | 108/108 | BQ1.2g tid+MTX10mg qw | MTX10mg qw | 12w | ①② |
| Zhao WH2018^[12]^ | 17/25 | 35-75 | 21/21 | FufangFSN1.5g tid | naproxen25mg qd-500mg bid | 12w | ①⑤ |
| Wang CD2011^[13]^ | 36/66 | / | 62/40 | FufangFSN1.0g tid/qid | GTW20mg tid | 16w | ①⑤⑥ |
| Meng JJ2016^[14]^ | 62/46 | 13-65 | 54/54 | FufangFSN1.05g tid | indomethacin50mg tid | 4w | ①③④⑤ |
| Gong CY2018^[15]^ | 32/28 | 18-68 | 30/30 | JGL500mg tid+ aceclofenac acid100mg bid+LEF20mg tid | FufangFSN1.5g tid+ aceclofenac acid100mg bid+LEF20mg qd | 4w | ① |
| Ju HY2015^[16]^ | 11/49 | / | 30/30 | JGL500mg bid+ aceclofenac acid100mg bid+LEF20mg qd | FufangFSN1.5g tid+ aceclofenac acid100mg bid+LEF20mg qd | 4w | ①③④⑥ |
| Ji Yl2010^[17]^ | 8/52 | / | 30/30 | KX600mg bid+MTX10mg qw | MTX10mg qw | 12w | ①③④⑤⑥ |
| Jia CP2016^[18]^ | 17/43 | 20-70 | 30/30 | KX600mg tid+MTX10-15mg qw + folic acid10mg qw +nimesulide100mg bid | MTX10-15mg qw+ folic acid 10mg qw +nimesulide100mg bid | 12w | ①②③④⑤⑥ |
| Lu J2011^[19]^ | 15/25 | 60-73 | 20/20 | KX300-600mg tid +MTX7.5mg qd | MTX7.5-15mg qw | 24w | ①②③④⑤⑥ |
| Tian Y2014^[20]^ | 13/47 | / | 30/30 | KX300mg tid+LEF10mg qd | MTX10mg qw+LEF10mg qd | 24w | ②⑥ |
| Zhang J2015^[21]^ | 28/44 | 30-67 | 36/36 | KX300mg tid+MTX10mg qw+diclofenac sodium75mg qd | MTX10mg qw+diclofenac sodium75mg qd | 12w | ①⑤⑥ |
| Lin CS2011^[22]^ | 37/122 | / | 80/79 | KX300-600mg tid | MTX10mg qw | 12w | ①②④⑤⑥ |
| Lu Y2018^[23]^ | 21/39 | 30-68 | 30/30 | KX600mg tid | MTX10-15mg qw | 24w | ①②⑤⑥ |
| Quan Y2018^[24]^ | / | 38-74 | 78/78 | KX300-600mg tid+ hydroxychloroquine sulfate200mg bid | hydroxychloroquine sulfate 200mg bid | 24w | ①②⑥ |
| Wang XD2011^[25]^ | 37/122 | / | 80/79 | KX600mg tid | MTX10-15mg qw | 12w | ①③④⑤⑥ |
| Lyu QW2013^[26]^ | 20/116 | / | 67/69 | GTW20mg tid | MTX7.5-12.5mg qw+folic acid10mg qw | 24w | ①② |
| Li JH 2017^[27]^ | 42/78 | 42-73 | 60/60 | GTW0.33-0.5mg/kg tid | MTX10mg qw+folic acid10mg qw | 12w | ①② |
| Feng J2017^[28]^ | 27/53 | 40-76 | 40/40 | GTW20mg tid+MTX2 qd | MTX5mg qd | 4w | ② |
| Liao ZH2016^[29]^ | 36/60 | / | 48/48 | GTW20mg tid+MTX10mg qw+ folic acid10mg qw | MTX10mg qw+folic acid10mg qw | 12w | ②③④⑤⑥ |
| Fan J2018^[30]^ | 43/47 | 28-70 | 45/45 | GTW30-60mg qd+MTX10mg qw | SASP1.0g bid+MTX10mg qw | 16w | ①② |
| Wang XF2015^[31]^ | 53/53 | / | 53/53 | GTW60mg qd+MTX10mg qw | MTX10mg qw | 12w | ①③④⑤⑥ |
| Gao DW2017^[32]^ | 24/56 | 28-72 | 40/40 | GTW20mg tid+MTX10mg qw+LEF10mg/d | MTX10mg qw+folic acid10mg qw | 12w | ①②③④⑤⑥ |
| Li GK2017^[33]^ | 45/35 | 44-67 | 40/40 | GTW10mg tid+MTX10mg qw | MTX10mg qw | 8w | ② |
| Yang XY2007^[34]^ | 34/86 | 19-68 | 60/60 | GTW20mg tid | MTX15mg qw | 4w | ①②④⑤⑥ |
| Yuan Y2018^[35]^ | 34/64 | 41-69 | 49/49 | GTW20mg tid+MTX10mg qw | MTX10mg qw | 12w | ①②⑥ |
| Zhao ML2017^[36]^ | 13/47 | / | 31/29 | GTW20mg tid+prednisone5-15mg qd+LEF20mg qd | prednisone5-15mgqd+LEF20mg qd | 24w | ①② |
| Wang HB2016^[37]^ | 21/29 | / | 25/25 | GTW20mg tid+MTX10mg qw | MTX10mg qw | 12w | ③④ |
| Li N2008^[38]^ | 13/47 | 38-78 | 30/30 | GTW10-20mg qd+SASP0.25-1.0g tid+diclofenac sodium25-75mg tid/celecoxib200mg bid | MTX10-15mg qw + SASP0.25-1.0g tid+diclofenac sodium25-75mg tid /celecoxib200mg bid | 4w | ②⑥ |
| Li LH2017^[39]^ | 15/45 | 19-68 | 30/30 | GTW20mg tid+LEF10mg qd+MTX10mg qw | LEF10mg qd+MTX10mg qw | 12w | ②⑥ |
| Wang YY2017^[40]^ | 34/62 | / | 48/48 | GTW20mg tid+MTX10mg qw+ folic acid10mg qw | MTX10mg qw+ folic acid10mg qw | 12w | ②③④⑤⑥ |
| Fu JB2001^[41]^ | 3/45 | 28-61 | 30/18 | GTW20mg tid+diclofenac sodium50mg tid | diclofenac sodium50mg tid | 12w | ③④⑤⑥ |
| Li NN2005^[42]^ | 19/31 | 40-78 | 25/25 | GTW20mg tid+NSAIDs | NSAIDs | 12w | ③④⑥ |
| Tan YZ2000^[43]^ | 15/60 | / | 35/40 | GTW20mg tid | MTX5-10mg qw+penicillamine125-250mg bid | 24w | ①②③④⑤⑥ |
| Zhang XL2012^[44]^ | 22/67 | 19-66 | 47/42 | GTW20mg tid+MTX10-15mg qw | MTX10-15mg qw | 12w | ①③④⑤⑥ |
| Yang M2013^[45]^ | 10/70 | 60-76 | 40/40 | GTW10mg tid+MTX10mg qw | MTX10mg qw | 24w | ①②③④⑤⑥ |
| Wang YQ2013^[46]^ | 56/70 | 30-61 | 76/50 | GTW10mg tid+MTX15mg qw | MTX15mg qw | 12w | ①② |
| Long H 2014^[47]^ | 21/27 | 60-73 | 24/24 | GTW20mg tid+LEF10mg/d | LEF10mg/d | 24w | ①③④⑥ |
| Shen J2002^[48]^ | 24/36 | 60-78 | 30/30 | GTW10mg tid+MTX7.5mg qw | MTX15mg qw | 12w | ①②⑤⑥ |
| Zhang R2011^[49]^ | 10/50 | 60-78 | 30/30 | GTW20mg tid+LEF20mg/d | LEF20mg/d | 24w | ②③④⑤⑥ |
| Sun DH 2003^[50]^ | 14/36 | / | 25/25 | GTW20mg tid+MTX10mg qw | MTX20mg qw+SASP0.25-1.0g bid | 12w | ①② |
| Xin XF2005^[51]^ | 18/72 | / | 30/60 | GTW10mg tid+MTX7.5-10mg qw | MTX7.5-10mg qw+diclofenac/naproxen | 48w | ①② |
| Chen ZF2018^[52]^ | 38/52 | 21-59 | 45/45 | GTW20mg tid+MTX10mg qw | MTX10mg qw | 12w | ①②⑥ |
| Zhuang MC2018^[53]^ | 24/74 | 41-73 | 49/49 | GTW0.33-0.5mg/kg tid+MTX10mg qw+folic acid10mg qw | MTX10mg qw+folic acid10mg qw | 24w | ①②③④⑤⑥ |
| Mo ML2018^[54]^ | 14/46 | 31-57 | 30/30 | GTW20mg tid+MTX10mg qw+methylprednisolone8mg/d | MTX10mg qw + methylprednisolone8mg/d | 12w | ②⑥ |
| Sun W2010^[55]^ | / | / | 40/40 | GTW20mg tid+MTX10mg qw | LEF20mg qd+MTX10mg qw | 24w | ①② |
| Lin WG2016^[56]^ | 39/61 | / | 50/50 | GTW10mg tid+MTX7.5mg qw +prednisone10mg qd +meloxicam15mg qd | MTX10mg qw+prednisone10mg qd+meloxicam15mg qd | 24w | ①② |
| Zhu JQ2016^[57]^ | / | / | 30/30 | GTW40mg/d+MTX10-15mg/w | LEF10mg/d+MTX10-15mg/w | 8w | ①⑤⑥ |
| Pan ZP2014^[58]^ | 5/35 | 20-61 | 20/20 | GTW20mg tid+MTX10mg qw | LEF20mg qd+MTX10mg qw | 12w | ①②③④⑤⑥ |
| Wu YJ2001^[59]^ | 15/55 | 42-74 | 35/35 | GTW10mg tid+MTX7.5mg qw +indomethacin100mg tid/ diclofenac25mg tid | MTX15mg qw +indomethacin100mg tid/diclofenac25mg tid | 12w | ①②③④⑤⑥ |
| Wang ML2018^[60]^ | / | 18-70 | 14/15 | GTW20mg tid | MTX7.5-12.5mg qw | 12w | ③④⑥ |
| Jiang M2015^[61]^ | 37/155 | / | 97/95 | GTW10mg tid+ SASP 500mg tid/1.0g bid | MTX 10mg qw+ SASP500mg tid/1.0g bid | 24w | ①② |
| Lv QW2014^[62]^ | 23/115 | 18-65 | 69/69 | GTW20mg tid | MTX7.5-12.5mg(0.20-0.25mg/kg)qw+folic acid10mg qw | 24w | ①②③④⑥ |
| Yang Z2011^[63]^ | 13/133 | 45-63 | 74/72 | LGT66μg tid | MTX10mg qw | 12w | ①②③④⑤ |
| Lei JH2013^[64]]^ | 42/62 | 48-79 | 52/52 | LGT12-24μg bid/tid +diclofenac sodium25-50mg tid | diclofenac sodium 25-50mg tid +MTX2-5mg/d | 24w | ①② |
| Tu SH2006^[65]^ | 12/62 | / | 38/36 | LGT66μg tid | MTX10mg qw | 12w | ①② |
| Su JS2010^[66]^ | 16/34 | 25-58 | 27/23 | QWTB10mL tid +nimesulide100mg bid | nimesulide100mg bid | 8w | ②④⑤⑥ |
| Li XL2015^[67]^ | 21/47 | 27-64 | 34/34 | QWTB10mL tid +MTX10mg qw | MTX10mg qw | 8w | ①④⑤⑥ |
| Jiang QZ2018^[68]^ | 23/37 | 37-58 | 34/26 | WB2.0g tid+MTX5-10mg qw | MTX5-10mg qw | 24w | ①② |
| Li SH2013^[69]^ | 20/80 | 28-69 | 50/50 | WB2.0gtid+MTX10mg qw | MTX10mg qw +LEF20mg qd | 12w | ①②③④⑤⑥ |
| Ji HW2013^[70]^ | / | 26-64 | 20/10 | WB2.0g tid | MTX10mg qw | 8w | ①②⑥ |
| Zhou T2009^[71]^ | 32/58 | 60-70 | 45/45 | YSJB8g tid+MTX10mg qw+diclofenac sodium75mg/d | MTX10mg qw +diclofenac sodium 75mg/d | 24w | ①②③④⑤⑥ |
| Ji HW 2016^[72]^ | 17/51 | 28-73 | 34/34 | YSJB8-12g tid+MTX7.5-15mg qw | MTX7.5-15mg qw+SASP2-3g/d | 12w | ① |
| Lao YQ2008^[73]^ | 28/52 | 18-65 | 40/40 | YSJB8g tid +MTX7.5-15mg qw +celecoxib200mg qd | MTX7.5-15mg qw +celecoxib200mg qd | 8w | ①③④⑤ |
| Zheng FZ2006^[74]^ | 13/47 | 20-69 | 40/20 | YSJB8g tid+MTX10mg qw+ folic acid10mg qw | GTW20mg bid+indomethacin25mg tid | 16w | ①②③④⑤⑥ |
| Bai MH2017^[75]^ | 40/80 | / | 60/60 | YSJB8-12g tid +MTX5-10mg qw/biw +LEF20-50mg/d+celecoxib200mg qd/100mg bid | MTX5-10mg qw/biw +LEF20-50mg/d+celecoxib200mg qd/100mg bid | 20w | ①②③⑤⑥ |
| Li XF2016^[76]^ | 59/21 | 18-65 | 40/40 | YSJB8-12g tid +LEF20-50mg qd | LEF20-50mg qd | 12w | ①②⑤⑥ |
| Li XF2015^[77]^ | 38/54 | / | 46/46 | YSJB8-12g tid +meloxicam7.5mg bid | meloxicam7.5mg bid | 16w | ①②⑤⑥ |
| Luo M2017^[78]^ | 43/73 | 15-56 | 58/58 | YSJB8g tid +MTX10-17.5mg qw +LEF20mg qd | MTX10-17.5mg qw+LEF20mg qd | 24w | ①⑥ |
| Li SP2008^[79]^ | 22/88 | / | 58/52 | YSJB8g tid+LEF20-50mg qd+MTX10mg qw | celecoxib200mg bid + LEF20-50mg qd +MTX10mg qw | 8w | ③④⑤⑥ |
| Wei Q 2012^[80]^ | 0/22 | 19-35 | 12/10 | YSJB8g tid+MTX10mg qw +LEF10mg qd | MTX10mg qw +LEF10mg qd | 24w | ① |
| Wu YJ2002^[81]^ | 13/57 | / | 40/30 | YXB1.50g tid | ZQFTN40-60mg tid | 8w | ①②⑥ |
| Gao M2015^[82]^ | 53/37 | 53-70 | 45/45 | YXB2.5g tid+diclofenac sodium75mg/d+MTX10mg qw | diclofenac sodium 75mg/d +MTX10mg qw | 8w | ①⑥ |
| Yu J2015^[83]^ | 39/73 | 31-75 | 56/56 | ZQFTN60mg bid +TGP600mg tid +MTX10-15mg qw | MTX10-15mg qw | 4w | ① |
| Xia L2016^[84]^ | 55/67 | / | 65/57 | ZQFTN60mg bid +celecoxib100mg bid | celecoxib100mg bid | 4w | ① |
| Huang ZS2010^[85]^ | 22/38 | 28-63 | 30/30 | ZQFTN60mg bid +MTX7.5mg qw | MTX15mg qw | 12w | ①②③④⑤⑥ |
| Zhu YM2017^[86]^ | 27/41 | / | 38/30 | ZQFTN60mg tid +MTX10mg qw +celecoxib100mg bid | MTX10mg qw +celecoxib100mg bid | 12w | ①②⑤⑥ |
| Ji H2006^[87]^ | 17/43 | 36-74 | 30/30 | ZQFTN20-40mg tid +MTX7.5mg qw | MTX15mg qw | 12w | ①②③④⑤⑥ |
| Huang GD2007^[88]^ | 66/130 | 26-60 | 100/96 | ZQFTN60mg tid | GTW0.33mg/kg tid | 12w | ①②⑤⑥ |
| Chen X2005^[89]^ | 20/62 | 16-68 | 52/30 | ZQFTN40mg tid | GTW20mg tid | 12w | ①②⑤⑥ |
| Wu YJ2003^[90]^ | 18/62 | 21-81 | 40/40 | ZQFTN40mg tid +MTX7.5mg qw | MTX15mg qw | 12w | ①②③④⑤⑥ |
| Fan CP2000^[91]^ | 50/79 | / | 88/41 | ZQFTN40mg tid | GTW20mg tid | 8w | ① |
| Yu YX2005^[92]^ | 11/61 | 15-69 | 38/34 | ZQFTN60mg tid +SASP0.5-1.0g bid | MTX10-15mg bid+SASP0.5-1.0g bid | 24w | ①②⑥ |
| Zhou B2014^[93]^ | 23/37 | 22-65 | 30/30 | ZQFTN60mg bid +MTX10mg qw | MTX10mg qw | 8w | ①③④⑤⑥ |
| Gu F2014^[94]^ | 23/67 | / | 45/45 | ZQFTN40mg tid +MTX10mg qw | MTX10mg qw | 24w | ①②⑤⑥ |
| Zhu YY2010^[95]^ | 37/97 | 25-78 | 67/67 | ZQFTN80-60mg bid + hydroxychloroquine sulfate100mg bid+MTX7.5mg qw | hydroxychloroquine sulfate100mg bid +MTX7.5mg qw | 240w | ①⑤⑥ |
| Wu HP2014^[96]^ | 7/53 | 19-69 | 30/30 | ZQFTN60mg bid +TGP600mg bid +MTX7.5mg qw +folic acid10mg qd | MTX7.5mg qw+folic acid10mg qd +meloxicam7.5mg bid/diclofenac25mg tid | 12w | ①③④⑤⑥ |
| Wang WQ2010^[97]^ | 66/120 | 11-73 | 120/66 | ZQFTN60mg bid +MTX10mg qw + folic acid20mg qw | MTX10mg qw+ folic acid20mg qw | 24w | ①②⑤⑥ |
| Yang CH2009^[98]^ | 29/91 | 18-69 | 60/60 | ZQFTN120mg bid+diclofenac sodium100mg qd +MTX10mg qw+ folic acid10mg qw | SASP500mg tid+diclofenac sodium100mg qd+MTX10mg qw+ folic acid10mg qw | 24w | ②⑤⑥ |
| Hu Q2010^[99]^ | 15/45 | / | 30/30 | ZQFTN60mg bid +LEF20mg qd +MTX10mg qw+ folic acid10mg qw | LEF20mg qd+MTX10mg qw+ folic acid10mg qw | 12w | ①②③④ |
| Yang SX2016^[100]^ | / | / | 15/15 | ZQFTN120mg bid+MTX10-15mg qw+LEF20mg/d | MTX10-15mg qw+LEF20mg/d | 12w | ①②③④⑤⑥ |
| Mo FQ2014^[101]^ | 24/74 | / | 49/49 | ZQFTN60mg tid+naproxen75mg bid+prednisone5-10mg qd +MTX10mg qw+SASP0.25-1.0g bid | naproxen75mg bid +prednisone 5-10mg qd +MTX10mg qw+SASP0.25-1.0g bid | 12w | ①③④⑤ |
| Li X2012^[102]^ | 17/63 | 29-65 | 40/40 | ZQFTN60mg bid+diclofenac sodium 75mg bid | GTW20mg bid+diclofenac sodium75mg bid | 12w | ②③④⑤⑥ |
| Xu CC2006^[103]^ | 47/113 | / | 80/80 | ZQFTN60mg bid+MTX10mg qw | penicillamine125mg tid +MTX10mg qw | 12w | ①②⑤⑥ |
| Li YP2008^[104]^ | 45/65 | / | 80/30 | ZQFTN40-80mg tid | GTW20-40mg tid | 12w | ①② |
| Yang DC2005^[105]^ | 13/47 | / | 30/30 | ZQFTN60mg bid | oxaprozin200mg qd | 8w | ①②③④⑤⑥ |
| Han WC2009^[106]^ | 19/61 | 18-76 | 42/38 | ZQFTN120mg bid+diclofenac sodiu100mg qd+MTX10mg qw+ folic acid10mg qw | diclofenac sodium100mg qd+MTX10mg qw+ folic acid10mg qw+SASP500mg tid | 24w | ①② |
| Zhang JL2015^[107]^ | 49/27 | 27-69 | 38/38 | ZQFTN120mg bid+MTX10mg qw | MTX10mg qw | 12w | ① |
| Yan JJ2012^[108]^ | 16/44 | 18-70 | 30/30 | ZQFTN60mg bid+MTX10mg qw+ folic acid10mg qw + hydroxychloroquine sulfate200mg bid | MTX10mg qw+ folic acid10mg qw+hydroxychloroquinesulfate200mg bid | 12w | ②③④⑥ |
| Liu YL2011^[109]^ | 22/58 | / | 40/40 | ZQFTN120mg bid+LEF20mg qd+meloxicam15mg/d | LEF20mg qd+meloxicam15mg/d | 12w | ①②⑥ |
| Li Q2014^[110]^ | 22/34 | 21-67 | 28/28 | ZQFTN60mg bid+prednisone5-10mg qd+SASP1g bid+MTX10mg qw+ folic acid10mg qw | prednisone5-10mg qd+SASP1g bid+MTX10mg qw+ folic acid10mg qw | 12w | ①②⑤ |
| Li YN2008^[111]^ | 25/44 | 17-67 | 35/34 | ZQFTN120mg bid+MTX10mg qw | MTX10mg qw | 8w | ①②⑤⑥ |
| Lu Y2011^[112]^ | 19/61 | 25-70 | 40/40 | ZQFTN120mg bid+MTX10mg qw+meloxicam7.5mg qd | MTX10mg qw+meloxicam7.5mg qd | 12w | ①②③④⑥ |
| Lin XJ2009^[113]^ | 22/58 | 28-68 | 40/40 | ZQFTN60mg bid+MTX10-15mg qw+SASP750mg tid | MTX10-15mg qw+SASP750mg tid | 12w | ①②③④⑤⑥ |
| He Y2010^[114]^ | 30/54 | 24-70 | 42/42 | ZQFTN120mg bid+SASP750mg tid +MTX10mg qw+meloxicam7.5mg qd | SASP750mg tid+MTX10mg qw+meloxicam7.5mg qd | 12w | ②⑥ |
| Zhang G2005^[115]^ | 90/90 | 16-68 | A60/B60/C60 | A:ZQFTN120mg bid | B:MTX15mg qw  C:GTW20mg tid | 12w | ①② |
| Zhang YB2017^[116]^ | 45/31 | 33-69 | 38/38 | ZQFTN60mg bid+MTX10mg qw+ folic acid10mg qw | MTX10mg qw+ folic acid10mg qw | 12w | ①⑥ |

Note: M: male; F:female; I: Intervention ; C: Comparison; BQ: Biqi capsule; FufangFSN: Fufang-Fengshining capsule/ tablet; JGL: Jingulian capsule; KX: Kunxian capsule; GTW: glucosides of Tripterygium Wilfordiitablet; LGT: Leigongteng tablet; QWTB: Qiweitongbi oral liquid; WB: Wangbi tablet; YSJB: Yishenjuanbi pill; YXB: Yuxuebi capsule/ tablet; ZQFTN: Zhengqingfengtongning tablet/ sustained-release tablet/ capsule; MTX:methotrexate; LEF: leflunomide; SASP: salazosulfapyridine; TGP: totalglucosidesofpaeony; NSAIDs: nonsteroidal anti-inflammatory drugs; ①:the clinical effectiveness rate; ②:the incidence of adverse drug reactions (ADRs); ③:joint tenderness; ④:joint swelling; ⑤:morning stiffnes; ⑥:erythrocyte sedimentation rate (ESR).

**Reference list of the included RCTs**

1. Liu W, Chen FY, Wang Y, Liu XY, Yang XY, Zhou YL, Yue M, Meng QG. Clinical observation on therapeutic effect of Biqi capsule and Zhengqingfengtongning pill in treating rheumatoid Arthritis Patients. China Journal of Traditional Chinese Medicine and Pharmacy, 2007,22(4):244-247.

2. Bai RX. Biqi Capsule for rheumatoid arthritis: A clinical multi-center andomized controlled trial.China Journal of Traditional Chinese Medicine and Pharmacy,2016,31(9):3821-3825.

3. Liu P. 48 Cases of Rheumatoid Arthritis Treated with Biqi Capsule. Capital Medicine,2012,19(20):53-54.

4. Jie HY, Wu QF, Ding ZX, Su X, Tang HY, Chen WW, Lu Y. Clinical study of Biqi capsule combined with methotrexate for treatment of rheumatoid arthritis. Chinese Journal of Integrated Traditional and Western Medicine, 2012, 32(2):195-198.

5. Jia Q, Yan P, Li ZZ, Yang JL. Observation on efficacy of Biqi capsules combined with low dose methotrexate in treatment of rheumatoid arthritis. Evaluation and Analysis of Drug-Use in Hospitals of China, 2018,18(3):361-363.

6. Wan HJ, Liu J, Xu JY, Fang HY. The efficacy of Biqi Capsules combined with low dose methotrexate in the treatment of rheumatoid arthritis. Chinese Journal of Biochemical Pharmaceutics,2012,33(5):669-671.

7. Dai LP. The clinical observation and nursing to Biqi Capsule in the treatment of rheumatoid arthritis. Home Medicine. 2016,(7):171-172.

8. Liu W, Zhang L, Xu Z. Clinical observation on treatment of rheumatoid arthritis with Biqi Capsule. Chinese Journal of Integrated Traditional and Western Medicine. 2006,26(2):157-159.

9. Hu XM. The clinical study on treatment of rheumatoid arthritis (Qi deficiency Blood stasis type) with BI QI Capsule. Henan University of Chinese Medicine,2006.

10. Bai HT, Li J. Clinical observation on 50 cases of Biqi capsule for treatment of rheumatoid arthritis. China Journal of Traditional Chinese Medicine and Pharmacy. 2009,24(11):1489-1490.

11. Yu WH. Clinical study on 216 cases of rheumatoid arthritis treated by traditional Chinese and western Medicine. Health Guide. 2017,38:87.

12. Zhao WH. Clinical study of Compound Fengfeining Capsule in the treatment of rheumatoid arthritis. Journal of China Prescription Drug. 2018,16(5):100.

13. Wang CD. Clinical study on the treatment of rheumatoid arthritis with Fufang Fengfeining tablets. Chinese Community Doctors. 2011,13(17):153-154.

14. Meng JJ. Clinical observation of Fufang Fengfeining tablets for treating rheumatoid arthritis. Journal of China Prescription Drug. 2016,14(6):3-4.

15. Gong CY, Ao HR, Wang MM, Li J. Clinical study of Jin Gu Lian capsule in the treatment of rheumatic obstruction syndrome rheumatoid arthritis. Chinese Journal of Clinical Rational Drug Use. 2018,11(16):18-19.

16. Ju HY. Jin Gu Lian capsule in the treatment of rheumatoid arthritis (RA) clinical study of rheumatism stagnation syndrome. Changchun University of Chinese Medicine, 2015.

17. Ji YL. A clinical study on the treatment of active rheumatism arthritis (RA) with the methotrexate (Amethopterin, MTX) and the Kunxian capsule. Guangzhou University of Chinese Medicine, 2010.

18. Jia CP. Clinical research on the treatment of rheumatoid arthritis with cold and dampness syndromes with the Kunxian capsule and the methotrexate. Hubei University of Chinese Medicine, 2016.

19. Lu J, Xu YJ, Tang DS. Efficacy and safety of Kunxian capsule combined with methotrexate in the treatment of elderly patients with rheumatoid arthritis. Journal of Chinese Medicinal Materials. 2011,34(5):835-837.

20. Tian Y. The clinical study on therapeutic effect of Kunxian capsule on active rheumatoid arthritis. Dalian Medical University, 2014.

21. Zhang J, Zhang WC. Clinical observation on 36 cases of rheumatoid arthritis treated with Kunxian capsule. Guiding Journal of Traditional Chinese Medicine and Pharmacy. 2015,21(11):69-70.

22. Lin CS, Yang XY, Dai L, Sun WF, Yang SF, Shen Y, Xu Q, Wang XD. Multi-center clinical study on therapeutic effect of Kunxian capsule on rheumatoid arthritis. Chinese Journal of Integrated Traditional and Western Medicine. 2011,31(6):769-774.

23. Lu Y. Clinical Efficacy of Kunxian Capsule in the treatment of rheumatoid arthritis. China Journal of Pharmaceutical Economics. 2018,13(8):78-80.

24. Quan Y, Wang J. Clinical study on Kunxian Capsules combined with hydroxychloroquine sulfate in treatment of rheumatoid srthritis. Drugs & Clinic. 2018,33(10):2638-2641.

25. Wang XD. Therapeutic effects research and contribution on IL-8 and γIP-10 of Kunxian capsule in treating rheumatoid arthritis. Guangzhou University of Chinese Medicine, 2011.

26. Lyu QW. Efficacy and safety of Tripterygium wilfordii Hook F versus methotrexate monotherapy in patients with rheumatoid arthritis: a muiticenter, randomized, controlled clinical trial. Peking Union Medical College, 2013.

27. Li JH, Gao J. Efficacy and safety of methotrexate (MTX) combined with Tripterygium wilfordii polyglycoside tablets in the treatment of rheumatoid arthritis. China Health Care & Nutrition. 2017,27(21):279-280.

28. Feng Q. Analysis of efficacy and adverse reactions on methotrexate combined with Tripterygium Wilfordii polyglycoside tablets in the treatment of rheumatoid arthritis. Chinese Journal of Modern Drug Application. 2017,11(16):131-132.

29. Liao ZH, Zhou ZH, Dai GD. Clinical effect of methotrexate and Leigong Teng Duo Dai Pian on treatment of rheumatoid arthritis. Chinese Journal of Biochemical and Pharmaceutics. 2016, 36(5):136-138.

30. Fan J. Therapeutic effect of methotrexate combined with Tripterygium wilfordii polyglycoside tablets in the treatment of rheumatoid arthritis. Zhejiang Clinical Medical Journal. 2018,20(5):834-835,838.

31. Wang XF, Luan ZJ .Clinical observation on the treatment of rheumatoid arthritis with Tripterygium glycosides and methotrexate. International Medicine and Health Guidance News. 2015,21(17):2598-2600.

32. Gao DW. The clinical efficacy and safety of methotrexate combined with low-does leflunomide and Tripterygium polyglycoside tablets in the treatment of rheumatoid arthritis. Journal of Taishan Medical College. 2017,38(5):524-525.

33. Li GK. Observation on the treatment effect of methotrexate combined with tripterygium glycoside in treating rheumatoid arthritis. Journal of Qiqihar University of Medicine. 2017,38(17):2007-2008.

34. Yang XY, Zhang L. Clinical observation on 60 cases of rheumatoid arthritis treated with Tripterygium polyglycoside tablets. Chinese Journal of Traditional Medical Science and Technology. 2007,14(2):130-131.

35. Yuan Y. Observation on the effect of Tripterygium wilfordii polyglycosides combined with methotrexate in the treatment of rheumatoid arthritis. Clinical Medicine. 2018,38(1):100-101.

36. Zhao ML. To observe the Clinical efficacy and safety of Tripterygium wilfordii glycosides tablets on active rheumatoid arthritis. Dalian Medical University, 2017.

37. Wang HB, Cui YH, Liu J. Effects of glucosidorum tripterygll totorum combined with methotrexate on rheumatoid arthritis. Chinese Journal of Clinical Rational Drug Use. 2016,9(35):52-53.

38. Li N, Ji H. Effects of Tripterygium Wilfordii unions SASP moves to rheumatoid arthritis. Journal of Liaoning University of Traditional Chinese Medicine. 2008,10(12):89-90.

39. Li LH, Mai PG, Chen BH. Effects of glucosidorum tripterygll totorum combined with immunosuppressor on rheumatoid arthritis and inflammatory factors. Modern Journal of Integrated Traditional Chinese and Western Medicine. 2017,26(10):1088-1090.

40. Wang YY, Jin MH, Wang WB. Glycosides of tripterygium wilfordii on the clinical intervention of rheumatoid arthritis. Chinese Journal of Biochemical and Pharmaceuticals. 2017, 37(4): 385-387.

41. Fu JB, Liang SX, Ren LQ, Li WW, Guo SJ. The effect of tripterygium glycosides on plasma TNF in patients with rheumatoid arthritis. The Journal of Traditional Chinese Orthopedics and Traumatology. 2001,13(9):13-14.

42. Li NN. The effect of tripterygium glycosides on plasma TNF-α and IL-6 in patients with rheumatoid arthritis. Journal of Guangxi Medical University. 2005,22(5):33-35.

43. Tan YZ, Wu CL, You YH. Clinical analysis of methotrexate combined with penicillamine in the treatment of rheumatoid arthritis. China Journal of Modern Medicine. 2000,10(12):71-72.

44. Zhang XL, Tan J. Clinical investigation of Tripterygium glycosides plus methotrexate for treatment of rheumatoid arthritis. Chinese Journal of Medicinal Guide. 2012,14(S2):602-603. doi:10.3969/j.issn.1009-0959.2012.z2.129.

45. Yang M, Zhou RH, Li BZ, Xu J, Shi YH, Mo HY. Clinical observation on treatment of rheumatoid arthritis with Tripterygium Wilfordii polyglycoside combined with methotrexate. Chinese Journal of Experimental Traditional Medical Formulae. 2013,19(17):300-304.

46. Wang YQ. Invistagtion of the efficacy of the combination of Tripterygium wilfordii with methotrexate treatmet on the rheumatoid arthritis. Chinese Journal of Primary Medicine and Pharmacy. 2013,20(11):1678-1680. doi:10.3760/cma.j.issn.1008-6706.2013.11.038.

47. Long H, Li R. Clinical observation of 48 cases of elderly-onset rheumatoid arthritis treating with Tripterygium glycoside combined with leflnomide. Contemporary Medicine. 2014,20(16):152-153.

48. Shen J, Zhang ZL. Clinical observation on the treatment of rheumatoid arthritis with Tripterygium Glycosides and low dosage methopterin. Zhejiang Journal of Integrated Traditional Chinese and Western Medicine. 2002,12(6):7-9.

49. Zhang R, Wu CL, Li SF, Hou P, Xiao WG. Clinical observation on treatment of rheumatoid arthritis with Tripterygium glycoside combined with leflnomide for active elderly-onset rheumatoid arthritis. Chinese Journal of Gerontology. 2011,31(12):2194-2196.

50. Sun DH, Shi Q. Clinical observation on slow-acting antirheumatic drugs combination on rheumatoid arthritis. Chinese Remedies & Clinics. 2003,3(3):203-204.

51. Xin XF,Chen Y. Integrated traditional Chinese and Western Medicine in the treatment of elderly patients with rheumatoid arthritis. Zhejiang Journal of Integrated Traditional Chinese and Western Medicine. 2005,15(5):292-293.

52. Chen ZF, Lan PM, Chen HY, Chen Y, Leng DY. Curative efficacy of Tripterygium Glycosides combined with methotrexate in treatment of rheumatoid arthritis active stage and its effects on serum CD62p and CD41 levels. Progress in Modern Biomedicine. 2018,18(20):3909-3912.

53. Zhuang MC, Wang XY, Chen PJ. Clinical efficacy of Tripterygium wilfordii polyglycoside tablets combined with methotrexate in the treatment of rheumatoid arthritis and its influence on inflammatory factors. China Medicine and Pharmacy. 2018,8(18):46-49.

54. Mo ML, Tang DX, Zhang J, Liu Y, Xie LH. A randomized controlled trial of combination therapy of methotrexate with iguratimod for patients with active rheumatoid arthritis. Journal of Fujian Medical University. 2018,52(4):245-248.

55. Sun W,Liu XM. Efficacy of methotrexate combined with leflunomide or Tripterygium wilfordii polyglycosides in the treatment of early rheumatoid arthritis with positive anti-cyclic citrullinated peptide antibody. Shanxi Medical Journal. 2010,39(1):59-60.

56. Lin WG, Lin QQ, Chen R. Effects observation of different treatment program in rheumatoid arthritis. China Modern Medicine. 2016,23(16):4-7.

57. Zhu JQ, Qian JD. Therapeutic effect of leflunomide combined with methotrexate in the treatment of rheumatoid arthritis. Journal of Frontiers of Medicine. 2016,6(4):192-193.

58. Pan ZP, Lin SP, Lin X. Observing the short-term curative effect of Tripterygium Glycosides combined with methotrexate in treating rheumatoid arthritis. Rheumatism and Arthritis. 2014,3(3):17-20.

59. Wu YJ, Lao ZY, Zhang ZL. Clinical observation on small doses Tripterygium Wilfordii polyglycoside combined with methotrexate in treating rheumatoid arthritis. Chinese Journal of Integrated Traditional and Western Medicine. 2001,21(12):895-896.

60. Wang M, Huang J, Fan H, He D, Zhao S, Shu Y, Li H, Liu L, Lu S, Xiao C. Treatment of rheumatoid arthritis using combination of methotrexate and Tripterygium Glycosides tablets-a quantitative plasma pharmacochemical and pseudotargeted metabolomic approach. Front Pharmacol. 2018; 9:1051. doi: 10.3389/fphar.2018.01051.

61. Jiang M, Zha Q, Zhang C, Lu C, Yan X, Zhu W, Liu W, Tu S, et al. Predicting and verifying outcome of Tripterygium wilfordii Hook F. based therapy in rheumatoid arthritis: from open to double-blinded randomized trial. Sci Rep. 2015; 5:9700. doi: 10.1038/srep09700.

62. Zhou YZ, Zhao LD, Chen H, Zhang Y, Wang DF, Huang LF, Lv QW, Liu B, Li Z, et al. Comparison of Tripterygium wilfordii Hook F with methotrexate in the treatment of active rheumatoid arthritis (TRIFRA): a randomised, controlled clinical trial. Annals of the Rheumatic Diseases. 2015;74:1078-1086.

63. Yang Z. The clinic effect of Tripterygium Wilforddi Hook on rheumatoid arthritis. China Pharmaceuticals. 2011,20(14):76-77.

64. Lei JH,Li XG. The Clinical Efficacy of Tripterygium wilfordii tablets in the treatment of rheumatoid arthritis. Gems of Health. 2013,12(4):352-352.

65. Tu SH, Hu YH. Assessment on life quality of patients with rheumatoid arthritis and efficacy of Tripterygium Wilfordii Hook. Journal of TCM Univ. of Hunan. 2006,26(2):25-27.

66. Su JS. The clinical observation on therapeutic effect of Qiwei Tongbi oral liquid combined with nimesulide in the treatment of rheumatoid arthritis. Journal of New Chinese Medicine. 2010,42(5):59-60.

67. Li XL, Deng GZ, Li J. Clinical observation on 34 cases of rheumatoid arthritis treated by Qiwei Tongbi Oral Liquid combined with conventional western medicine. Guiding Journal of Traditional Chinese Medicine and Pharmacy. 2015,21(3):69-71.

68. Jiang QZ, Zhang HT. To observe the clinical effect of Qibi Tablet in the treatment of rheumatoid arthritis. World Latest Medicine Information. 2018,18(49):162.

69. Li SH. Observation on the curative effect of Wangbi tablet combined with methotrexate in the treatment of rheumatoid arthritis. Liaoning Journal of Traditional Chinese Medicine. 2013,40(2):297-298.

70. Ji HW. Clinical observation of Wangbi Tablet in the treatment of rheumatoid arthritis (Deficiency of liver and kidney combined with blood stasis and blockage syndrome). Rheumatology Professional Committee of Chinese Association of Integrative Medicine. Compilation of the 11th National Conference on Integrative Medicine and Rheumatology,2013:5.

71. Zhou T. Study of Yishen Juanbi pills combined with methotrexate on elderly onset rheumatoid arthritis. Chinese Journal of Clinical Rational Drug Use. 2009,2(8):13-15.

72. Yue SL. A Study on the therapeutic effect of methamidopterin combined with Yishen Juanbi Pill on rheumatoid arthritis .Journal of Frontiers of Medicine. 2016,6(4):51-52.

73. Lao YQ, Zhang JP, Zhang B, Jiang JY, Liao YQ, Yu XF ,Zhou CY. Effect of Yishen Juanbi Pill on serum PAF content in patients with rheumatoid arthritis in active stage. Journal of New Chinese Medicine. 2008,40(10):32-33.

74. Zheng FZ, Meng QL. Clinical observation on Yishen Juanbi Pill combined with methotrexate in the treatment of rheumatoid arthritis. Branch Committee of Rheumatology of China Association of Chinese Medicine. The 11th National Symposium on Traditional Chinese Medicine Rheumatology,2006:2.

75. Bai MH, Cheng L, Zhang ZQ. Therapeutic effect of Yishen Juanbi pill combined with antirheumatic drugs for treating rheumatoid arthritis. Shaanxi Journal of Traditional Chinese Medicine. 2017,38(7):932-933.

76. Li XF, Zhong ST, Deng SM. Clinical research on rheumatoid arthritis treated with Kidney-Benefiting and Arthritis-Eliminating Pill combined with leflunomide tablets. Henan Traditional Chinese Medicine. 2016,36(11):2007-2009.

77. Li XF, Zhong ST, Deng SM. Therapeutic effect of Yishen Juanbi Pill combined with meloxicam on active rheumatoid arthritis. Guiding Journal of Traditional Chinese Medicine and Pharmacy. 2015,21(17):81-83.

78. Luo M. Clinical observation of Yishen Juanbi Pill in the treatment of rheumatoid arthritis. Nei Mongol Journal of Traditional Chinese Medicine. 2017,36(19):23-24.

79. Li SP, Hu XM, Jin ZM. Effects of Yishen Juanbi Pill in the treatment of rheumatoid arthritis. Zhejiang Clinical Medical Journal. 2008,10(5):628-629.

80. Wei Q, Feng YG, Liu XJ, Wang JL, Meng FT, Cui XC. 12 Cases of pre-pregnancy rheumatoid arthritis treated with Yishen Juanbi pill.Traditional Chinese Medicinal Research. 2012,25(12):39-40.

81. Wu YJ, Shen J, Zhang ZL. Clinical study on treatment of rheumatoid arthritis with Yu Xue Bi capsule. Chinese Journal of Information on TCM. 2002,9(2):13-14.

82. Gao M, Chen L, Li T, Tang ZX. Clinical observation of Yuxuebi tablet in the treatment of rheumatoid arthritis. Modern Diagnosis and Treatment. 2015,26(3):678-679.

83. Yu J, Xie QB, Bao Y, Ma L.Total glucosides of paeony capsule combined with Zhengqing Fengtongning tablet in treatment of rheumatoid arthritis. Medical Journal of West China. 2015,27(7):992-995.

84. Xia L, Xu YB, Li P, Huang LK. Efficacy of celecoxib combined with Zhengqingfengtongning tablets in the treatment of rheumatoid arthritis. Journal of North Sichuan Medical College. 2016,31(5):720-722.

85. Huang ZS,Wang N. Clinical observation of Zhengqing Fengtongning sustained release tablets in the treatment of rheumatoid arthritis. Hebei Medical Journal. 2010,32(1):59-60.

86. Zhu YM, Xu L. The effects of Zhengqing Fengtongning tablets, methotrexate and NSAIDs on rheumatoid arthritis. Health Way. 2017,16(11):54-55.

87. Ji H,Zhu ZH. Low-dose Zhengqing Fengtongning tablets combined with methotrexate in the treatment of 30 cases with rheumatoid arthritis. Herald of Medicine. 2006,25(1):37-38.

88. Huang GD, Li JB, Huang YH, Huang DF, Tang LJ, Zhang S, Li Q.The clinical study on sinomenine for 100 patients with rheumatoid arthritis. Journal of Emergency in Traditional Chinese Medicine. 2007,16(4):416-417.

89. Chen X, Li H. Clinical Study on Zhengqing Fengtongning tablets in the treatment of rheumatoid arthritis. Journal of Anhui Traditional Chinese Medical College. 2005,24(1):9-11.

90. Wu YJ. Clinical observation on 40 cases of rheumatoid arthritis treated by low dose methotrexate and Zhengqing Fengtongning tablets. Journal of New Chinese Medicine. 2003,35(1):40-41.

91. Fan CP, Chen QQ, Qi WL. Comparison of therapeutic effects of Zhengqing Fengtongning and Tripterygium wilfordii polyglycosides on rheumatoid arthritis. China Pharmaceuticals. 2000,9(10):49.

92. Yu YX,Chi SH. Therapeutic effect of Zhengqing Fengtongning combined with sulfasalazine on rheumatoid arthritis. Clinical Medicine of China. 2005,21(9):808-809.

93. Zhou B, Zhou YL, Lin WM, Shi P, Fu Y, Cao JY. Effect of sinomenine combined with methotrexate on the expression of IL-1,IL-17 and TNF-α in rheumatoid arthritis patients. Modern Journal of Integrated Traditional Chinese and Western Medicine. 2014,23(19):2066-2068.

94. Gu F, Sun Y, Chen SW, Wang P, Ding CZ, Sun LY. Clinical study of sinomenine in combination with methotrexate for the treatment of active rheumatoid arthritis. Shanghai Journal of Traditional Chinese Medicine. 2014,48(6):58-60.

95. Zhu YY, Ling X. 67 Cases with rheumatoid arthritis treated with Zhengqing Fengtongning and Western Medicine. Shaanxi Journal of Traditional Chinese Medicine. 2010,31(12):1615-1617.

96. Wu HP,Wang YQ. Total glucosides of paeony combined with total glucosides of paeony combined with Zhengqing Fengtongning treat RA(Rheumatoid Arthritis) in active stage. Journal of Zhejiang Chinese Medical University. 2014,38(4):422-425.

97. Wang WQ. Methotrexate combined with Zhengqing Fengtongning sustained-release tablets in the treatment of 120 cases with rheumatoid arthritis.Zhejiang Practical Medicine. 2010,15(4):280-281.

98. Yang CH. The Effect Observation of methotrexate combined with traditional Chinese drug in treatment with rheumatoid arthritis. Chinese Primary Health Care. 2009,23(10):130-131.

99. Hu Q, Guan XH, Lian Y, Lei LH, Tao L. Therapeutic effect observations of leflunomi de and methopterin combined with Zhengqing Fengtongning in treatment of rheumatoid arthritis. Journal of Modern Clinical Medicine. 2010,36(5):338-340.

100. Yang SX. Clinical study of sinomenine combined with methotrexate in treating rheumatoid arthritis. Guangzhou University of Chinese Medicine, 2016.

101. Mo FQ, Cai YX. Pharmacological and clinical observation of Zhengqing Fengtongning sustained release tablets on rheumatoid arthritis. China Health Care & Nutrition. 2014,(1):403-403.

102. Li X. Clinical observation on 40 Cases of rheumatoid arthritis rreated by Zhengqing Fengtongning sustained release tablets. Guiding Journal of Traditional Chinese Medicine and Pharmacy. 2012,18(3):79-80.

103. Xu CC. Clinical observation on Zhengqing-Fengtongnin tablets for the treatment of 80 cases of rheumatoid arthritis. Guiding Journal of TCM. 2006,12(11):33-35.

104. Li YP. Therapeutic effect of Zhengqing Fengtongning sustained-release tablets for treating rheumatoid arthritis. The Journal of Traditional Chinese Orthopedics and Traumatology. 2008,20(4):50.

105. Yang DC, Zheng XC. To explore the curative effect of Zhengqing Fengtongning sustained-release tablets on rheumatoid arthritis. Drug Evaluation. 2005,2(2):152-153.

106. Han WC, Huang Y. The effect of ZhengQinFengTongNing for rheumatoid arthritis therapy combined with methotrexate. Medical Journal of West China. 2009,21(5):766-768.

107. Zhang JL. Clinical observation of Zhengqing Fengtongning combined with methotrexate on rheumatoid arthritis. Chinese Journal of Modern Drug Application. 2015,9(7):110-111.

108. Yan JJ. Zhengqing Fengtongning combined with methotrexate treatment of rheumatoid arthritis clinical efficacy studies. Hubei University of Chinese Medicine,2012.

109. Liu YL .The treatment of active rheumatoid arthritis using the method of Zhengqing Fengtongning retard tablets combined with leflunomide. Hebei Medical University,2011.

110. Li Q, Zhu Y. A randomized controlled study of treatment effectiveness of Zhengqing Fengtongning added up with methotrexate therapy in rheumatoid arthritis. Journal of Practical Traditional Chinese Internal Medicine. 2014,28(7):86-88.

111. Li YN, Feng XG, Zhang SL, Li CM. Effects of sinomenine combined with methotrexate on rheumatoid arthritis. Practical Pharmacy and Clinical Remedies. 2008,11(5):285-287.

112. Lu Y,Su JM. The Clinical Efficacy of Zhengqing Fengtongning combined with methotrexate in the treatment of rheumatoid arthritis. Journal of Traditional Chinese Medicine. 2011,38(10):2019-2021.

113. Lin XJ, Cai XY, Ye JH. The clinical observation on sinomenine for rheumatoid arthritis .Journal of TCM Univ. of Hunan. 2009,29(8):52-54.

114. He Y. Clinical observation of Zhengqing fengtong ning for disease activity of rheumatic arthritis. Chongqing Medicine. 2010,39(12):1555-1556.

115. Zhang G, Fu ZL, Wang K, Liu XM. The study of Zhengqinfengtongning on expression of chemokine RANTES in patients with rheumatoid arthritis. Proceeding of Clinical Medicine. 2005,14(12):893-895.

116. Zhang YB, Zhao R. The effect of Zhengqingfengtongning combined with methotrexate on adjuvant therapy in patients with rheumatoid arthritis and its effect on laboratory indexes. Chinese and Foreign Medical Research. 2017,15(32):11-12.

**Table S3.** Results of the network meta-analysis for joint tenderness (upper-right quadrant) and the joint swelling (lower-left quadrant).

| BQ | -0.58  (-7.73,7.13) | 1.66  (-12.85,18.07) | 0.45  (-7.07,8.08) | 0.66  (-3.33,4.67) | -1.26  (-17.01,12.64) | -- | -0.35  (-10.99,10.52) | 2.23  (-3.37,7.93) | -0.34  (-4.15,3.19) | -0.91  (-4.58,2.76) |
| --- | --- | --- | --- | --- | --- | --- | --- | --- | --- | --- |
| -0.53  (-7.30,6.59) | FufangFSN | 2.389(-10.48,15.92) | 2.39  (-10.48,15.92) | 2.39  (-10.48,15.92) | 2.39  (-10.48,15.92) | -- | 0.20  (-11.67,11.57) | 2.76  (-4.56,10.05) | 0.10  (-6.69,6.88) | -0.45  (-6.83,5.93) |
| -1.67  (-16.84,14.64) | -1.15  (-15.06,13.35) | JGL | -1.57  (-17.28,14.53) | -1.11  (-16.29,12.95) | -3.30  (-23.65,16.59) | -- | -2.14  (-20.32,14.73) | 0.33  (-15.22,15.22) | -2.24  (-17.4,12.07) | -2.71  (-17.74,11.38) |
| -0.21 (-6.09,5.88) | 0.22  (-7.34,7.89) | 1.44  (-15.06,17.20) | KX | 0.23  (-6.76,7.03) | -1.58  (-18.81,14.35) | -- | -0.68  (-13.22,10.87) | 1.86  (-5.85,9.52) | -0.86  (-7.86,6.10) | -1.37  (-8.09,5.17) |
| 0.93  (-2.96,4.91) | 1.38  (-4.91,7.53) | 2.59  (-13.46,17.53) | 1.15  (-3.95,5.98) | GTW | -1.96  (-17.89,12.06) | -- | -1.03  (-11.34,8.83) | 1.64  (-2.66,5.78) | -1.09  (-3.66,1.65) | -1.58  (-3.21,0.16) |
| 0.076  (-13.51,13.08) | 0.47  (-14.12,14.78) | 1.38  (-18.88,21.40) | 0.21  (-14.23,14.00) | -0.84  (-14.54,12.17) | LGT | -- | 0.93 (-16.67,19.52) | 3.61  (-11.38,19.79) | 0.94  (-13.25,16.58) | 0.41  (-13.54,15.89) |
| -1.93  (-6.34,2.60) | -1.51  (-8.20,4.95) | -0.30  (-16.51,14.85) | -1.75  (-7.32,3.59) | -2.87  (-6.01,0.24) | -2.09  (-15.23,11.71) | QWTB | -- | -- | -- | -- |
| -4.47  (-12.56,4.13) | -3.98  (-13.48,5.59) | -2.65  (-20.43,13.62) | -4.16  (-12.98,4.75) | -5.35  (-12.89,2.43) | -4.47  (-19.66,11.09) | -2.46  (-10.36,5.55) | WB | 2.64  (-7.74,13.13) | -0.020  (-9.96,10.20) | -0.55  (-10.30,9.49) |
| -0.38  (-5.73,5.02) | 0.085  (-7.07,7.23) | 1.32  (-14.84,16.56) | -0.17  (-6.48,5.99) | -1.28  (-5.47,2.74) | -0.39  (-13.82,13.67) | 1.61  (-3.35,6.36) | 3.99  (-4.57,12.57) | YSJB | -2.71  (-7.07,1.77) | -3.22  (-7.15,0.97) |
| -0.15  (-3.89,3.52) | 0.31  (-6.15,6.42) | 1.47  (-14.64,16.37) | 0.059  (-5.26,4.84) | -1.08  (-3.49,1.11) | -0.26 (-13.14,13.32) | 1.79  (-1.52,4.91) | 4.23  (-3.63,11.80) | 0.19  (-4.12,4.55) | ZQFTN | -0.52  (-2.68,1.60) |
| -0.31  (-3.85,3.34) | 0.13  (-5.99,6.06) | 1.34  (-14.74,16.19) | -0.11  (-4.97,4.45) | -1.26  (-2.90,0.36) | -0.45  (-13.30,13.17) | 1.63  (-1.07,4.32) | 4.09  (-3.55,11.49) | 0.0027  (-3.86,4.07) | -0.17  (-1.84,1.68) | CM |

**Table S4.** Results of the network meta-analysis for morning stiffness (upper-right quadrant) and ESR (lower-left quadrant).

| BQ | 0.50  (-54.67,57.42) | -- | -13.98  (-58.84,31.31) | 1.38  (-40.1,47.19) | 3.54  (-112.7,121.5) | -4.67  (-52.03,46.28) | -10.12  (-107.9,115.2) | 2.53  (-60.98,67.55) | -- | 3.64  (-35.39,44.33) | -8.30  (-47.83,34.86) |
| --- | --- | --- | --- | --- | --- | --- | --- | --- | --- | --- | --- |
| 6.84  (-53.26,69.08) | FufangFSN | -- | -14.72  (-64.89,34.22) | 1.80  (-43.91,44.14) | 3.08  (-121.3,127.1) | -4.82  (-54.53,42.31) | -9.60  (-122.3,108.0) | 2.53  (-62.88,73.39) | -- | 3.31  (-43.32,45.1) | -8.60  (-52.92,32.0) |
| 9.66  (-64.5,92.09) | 3.91  (-47.27,53.89) | JGL | -- | -- | -- | -- | -- | -- | -- | -- | -- |
| -5.88  (-27.41,15.42) | -13.07  (-77.25,49.68) | -15.34  (-100.8,61.21) | KX | 16.00  (-14.18,48.32) | 18.86  (-104,142.2) | 9.95  (-26.74,45.13) | 4.12  (-98.65,127.8) | 17.41  (-41.75,74.81) | -- | 17.89  (-11.27,45.76) | 6.04  (-19.71,31.8) |
| -4.47  (-16.58,8.42) | -11.41  (-72.17,49.22) | -14.03  (-95.38,60.78) | 1.71  (-18.06,19.73) | GTW | 1.30  (-118.2,121.1) | -6.40  (-36.64,23.9) | -12.48  (-115.2,110.4) | 0.34  (-50.63,52.39) | -- | 1.42  (-15.57,20.52) | -10.12  (-25.89,5.30) |
| -- | -- | -- | -- | -- | LGT | -7.72  (-130.7,114.5) | -13.14  (-173.9,150.4) | -1.12  (-138.3,139.4) | -- | 0.14  (-119.6,121.3) | -12.02  (-130.2,109) |
| 2.23  (-25.02,28.82) | -4.81  (-72.77,62.2) | -7.91  (-94.14,72.26) | 7.83  (-21.86,37.93) | 6.38  (-18.11,30.46) | -- | QWTB | -5.74  (-111,112.70) | 7.08  (-51.38,65.74) | -- | 7.57  (-21.24,37.5) | -3.66  (-30.86,22.58) |
| -5.17  (-50.17,37.43) | -11.59  (-83.98,57.73) | -15.75  (-103,65.64) | 0.60  (-45.10,44.94) | -0.95  (-43.45,42.11) | -- | -7.05  (-56.26,40.83) | WB | 11.99  (-113.9,130.1) | -- | 13.28  (-109.2,115.7) | 1.56  (-119.4,104.9) |
| -3.64  (-26.89,19.97) | -10.34  (-76.17,53.93) | -14.13  (-97.65,62.17) | 1.46  (-24.93,29.2) | 0.56  (-20.59,20.96) | -- | -5.94  (-39.46,26.73) | 1.12  (-45.87,50.02) | YSJB | -- | 0.75  (-51.34,52.22) | -10.33  (-61.91,39.07) |
| -16.23  (-39.48,7.602) | -23.55  (-87.59,42.74) | -26.02  (-112.7,52.44) | -10.12  (-39.26,19.53) | -11.74  (-35.90,11.30) | -- | -18.12  (-51.46,14.68) | -10.90  (-59.54,38.84) | -12.06  (-42.25,18.48) | YXB | -- | -- |
| -7.33  (-17.16,1.72) | -14.47  (-76.18,45.29) | -17.12  (-99.09,56.99) | -1.41  (-22.24,17.47) | -3.10  (-11.91,5.99) | -- | -9.30  (-34.72,15.66) | -1.89  (-43.62,40.96) | -3.67  (-25.96,18.38) | 8.82  (-12.92,31.08) | ZQFTN | -11.77  (-24.08,0.86) |
| -10.18  (-22.26,2.13) | -17.36  (-77.85,43.56) | -19.62  (-101.4,54.98) | -3.91  (-23.23,13.42) | -5.59  (-9.85,-2.21) | -- | -12.15  (-35.91,11.81) | -4.85  (-47.17,37.95) | -6.27  (-26.39,14.54) | 6.00  (-17.06,29.94) | -2.52  (-10.88,6.031) | CM |

**Table S5.** Risk-of-bias judgements for the included RCTs (RoB 2)

| Study ID | Bias arising from the randomization process | Bias due to deviations from intended interventions | Bias due to missing outcome data | Bias in measurement of the outcome | Bias in selection of the reported result | Overall bias |
| --- | --- | --- | --- | --- | --- | --- |
| Liu W2007 | Low | Low | Low | Low | Low | Low |
| Bai RX2016 | Low | Low | Low | Low | High | High |
| Liu P2012 | High | Low | Low | Low | Low | High |
| Jie HY2012 | High | Low | Low | Low | Low | High |
| Jia Q2018 | Low | Low | Low | Low | Low | Low |
| Wan HJ2012 | Low | Low | Low | Low | Low | Low |
| Dai LP2016 | Low | Low | Low | Some concerns | Low | Some concerns |
| Liu W2006 | Low | Low | Low | Low | Some concerns | Some concerns |
| Hu XM2006 | Low | Low | Low | Low | Low | Low |
| Bai HT2009 | Low | Low | Low | Low | Some concerns | Some concerns |
| Yu WH2017 | Some concerns | Low | Low | Low | Some concerns | Some concerns |
| Zhao WH2018 | Some concerns | Low | Low | Low | High | High |
| Wang CD2011 | Some concerns | Low | Low | Low | High | High |
| Meng JJ2016 | Some concerns | Low | Low | Low | Some concerns | Some concerns |
| Gong CY2018 | Some concerns | Low | Low | Low | Low | Some concerns |
| Ju HY2015 | High | Low | Low | Low | Low | High |
| Ji Yl2010 | Low | Low | Low | Low | Low | Low |
| Jia CP2016 | Some concerns | Low | Low | Low | Low | Some concerns |
| Lu J2011 | Some concerns | Low | Low | Low | Low | Some concerns |
| Tian Y2014 | Low | Low | Low | Low | High | High |
| Zhang J2015 | Some concerns | Low | Low | Low | Low | Some concerns |
| Lin CS2011 | Low | Some concerns | Low | Low | Low | Some concerns |
| Lu Y2018 | Low | Low | Low | Low | Low | Low |
| Quan Y2018 | Some concerns | Low | Low | Low | Low | Some concerns |
| Wang XD | Some concerns | Some concerns | Low | Low | Low | Some concerns |
| Lyu QW2013 | Low | Some concerns | Low | Low | Low | Some concerns |
| Li JH 2017 | Low | Low | Low | Low | Low | Low |
| Feng J2017 | Some concerns | Low | Low | Low | Low | Some concerns |
| Liao ZH2016 | Low | Low | Low | Low | Low | Low |
| Fan J2018 | Some concerns | Low | Low | Low | Low | Some concerns |
| Wang XF2015 | Low | Low | Low | Low | Low | Low |
| Gao DW2017 | Low | Low | Low | Low | Low | Low |
| Li GK2017 | Low | Low | Low | Low | Low | Low |
| Yang XY2007 | Some concerns | Low | Low | Some concerns | Low | Some concerns |
| Yuan Y2018 | Some concerns | Low | Low | Low | Low | Some concerns |
| Zhao ML2017 | Some concerns | Some concerns | Some concerns | Low | Low | Some concerns |
| Wang HB2016 | Some concerns | Low | Low | Low | Low | Some concerns |
| Li N2008 | Some concerns | Low | Low | Low | High | High |
| Li LH2017 | Some concerns | Low | Low | Low | Low | Some concerns |
| Wang YY2017 | Some concerns | Low | Low | Low | Low | Some concerns |
| Fu JB2001 | High | Low | Low | Low | Low | High |
| Li NN2005 | Low | Low | Low | Low | Low | Low |
| Tan YZ2000 | Some concerns | Low | Low | Low | High | High |
| Zhang XL2012 | Some concerns | Low | Low | High | Low | High |
| Yang M2013 | Some concerns | Low | Some concerns | Low | Low | Some concerns |
| Wang YQ2013 | Low | Low | Low | Low | Low | Low |
| Long H 2014 | Some concerns | Low | Low | Low | Low | Some concerns |
| Shen J2002 | Some concerns | Low | Low | Low | Low | Some concerns |
| Zhang R2011 | Some concerns | Low | Low | Low | Low | Some concerns |
| Sun DH 2003 | Some concerns | Low | Low | Low | Some concerns | Some concerns |
| Xin XF2005 | Some concerns | Low | Low | Low | Low | Some concerns |
| Chen ZF2018 | Low | Low | Low | Low | Low | Low |
| Zhuang MC2018 | Low | Low | Low | Low | Low | Low |
| Mo ML2018 | Low | Low | Low | Low | Some concerns | Some concerns |
| Sun W2010 | Some concerns | Low | Low | Low | Low | Some concerns |
| Lin WG2016 | High | Low | Low | Low | Low | High |
| Zhu JQ2016 | Some concerns | Low | Low | Low | Low | Some concerns |
| Pan ZP2014 | Low | Low | Low | Low | Low | Low |
| Wu YJ2001 | Some concerns | Low | Low | Low | Low | Some concerns |
| Wang ML2018 | Some concerns | Low | Low | Low | Low | Some concerns |
| Jiang M2015 | Low | Low | Low | Low | Low | Low |
| Lv QW2014 | Low | Low | Low | Low | Low | Low |
| Yang Z2011 | Some concerns | Low | Low | Low | Low | Some concerns |
| Lei JH2013 | High | Low | Low | Low | Low | Some concerns |
| Tu SH2006 | Some concerns | Low | Some concerns | Low | High | High |
| Su JS201 | Some concerns | Low | Low | Low | Low | Some concerns |
| Li XL2015 | Low | Low | Low | Low | Low | Low |
| Jiang QZ2018 | Low | Low | Low | Low | Low | Low |
| Li SH2013 | Some concerns | Low | Low | Low | Low | Some concerns |
| Ji HW2013 | High | Some concerns | Low | Low | Low | High |
| Zhou T2009 | Some concerns | Low | Low | Low | Low | Some concerns |
| Ji HW 2016 | Some concerns | Low | Low | High | Low | High |
| Lao YQ2008 | Some concerns | High | High | Low | Low | High |
| Zheng FZ2006 | Some concerns | Low | Low | Low | Low | Some concerns |
| Bai MH2017 | Low | Low | Low | Low | High | High |
| Li XF2016 | Some concerns | Low | Low | Low | Low | Some concerns |
| Li XF2015 | High | Low | Low | Low | Low | High |
| Luo M2017 | Some concerns | Low | Low | Low | Low | Some concerns |
| Li SP2008 | Some concerns | Low | Low | Low | Low | Some concerns |
| Wei Q 2012 | Low | Low | Low | Low | Low | Low |
| Wu YJ2002 | High | Low | Low | Low | High | High |
| Gao M2015 | Low | Low | Low | Low | Low | Low |
| Yu J2015 | Low | Low | Low | Low | Low | Low |
| Xia L2016 | Some concerns | Low | Low | Low | Low | Some concerns |
| Huang ZS2010 | High | Low | Low | Low | Low | High |
| Zhu YM2017 | Some concerns | Low | Low | Low | Low | Some concerns |
| Ji H2006 | Some concerns | Low | Low | Low | High | High |
| Huang GD200 | Low | Low | Low | Low | Low | Low |
| Chen X200 | Some concerns | Low | Low | Low | High | High |
| Wu YJ200 | Some concerns | Low | Low | Low | Low | Some concerns |
| Fan CP2000 | High | High | High | Low | Low | High |
| Yu YX2005 | High | Low | Low | Low | High | High |
| Zhou B2014 | Some concerns | Low | Low | Low | High | High |
| Gu F2014 | Low | Low | Low | Low | Low | Low |
| Zhu YY2010 | Low | Low | Low | Low | Low | Low |
| Wu HP2014 | Some concerns | Low | Low | Low | Low | Some concerns |
| Wang WQ2010 | High | Low | Low | Low | Low | High |
| Yang CH2009 | Some concerns | Low | Low | Low | Low | Some concerns |
| Hu Q2010 | Low | Low | Low | Low | High | High |
| Yang SX2016 | Low | Low | Low | Low | Low | Low |
| Mo FQ2014 | Some concerns | Low | Low | Low | Low | Some concerns |
| Li X2012 | High | Low | Low | Low | Low | High |
| Xu CC2006 | High | Low | Low | Low | Low | High |
| Li YP2008 | Low | Low | Low | Low | Low | Low |
| Yang DC2005 | High | Low | Low | Low | High | High |
| Han WC2009 | High | Low | Low | Low | High | High |
| Zhang JL2015 | Some concerns | Low | Low | Low | Low | Some concerns |
| Yan JJ2012 | Some concerns | Low | Low | Low | Low | Some concerns |
| Liu YL2011 | Some concerns | Low | Low | Low | Low | Some concerns |
| Li Q2014 | High | Low | Low | Low | High | High |
| Li YN2008 | Some concerns | Low | Low | Low | Low | Some concerns |
| Lu Y2011 | Some concerns | Low | Low | Low | Low | Some concerns |
| Lin XJ2009 | Some concerns | Low | Low | Low | Low | Some concerns |
| He Y2010 | Some concerns | Low | Low | Low | Low | Some concerns |
| Zhang G2005 | Some concerns | Low | Low | Low | High | High |
| Zhang YB2017 | High | Low | Low | Low | Low | High |
